# Effect of SGLT-2 inhibitors on arrhythmia events: insight from an updated secondary analysis of > 80,000 patients (the SGLT2i—Arrhythmias and Sudden Cardiac Death)

**DOI:** 10.1186/s12933-024-02137-x

**Published:** 2024-02-24

**Authors:** Jia Liao, Ramin Ebrahimi, Zhiyu Ling, Christian Meyer, Martin Martinek, Philipp Sommer, Piotr Futyma, Davide Di Vece, Alexandra Schratter, Willem-Jan Acou, Lin Zhu, Márcio G. Kiuchi, Shaowen Liu, Yuehui Yin, Helmut Pürerfellner, Christian Templin, Shaojie Chen

**Affiliations:** 1 Department of Cardiology, The Second Affiliated Hospital of Chongqing Medical University, Chongqing, China; 2 Department of Cardiology, Union Hospital, Tongji Medical College, Huazhong University of Science and Technology, Wuhan, China; 3 Heart Clinic Pratteln, Zentrum Für Kardiologie, Pratteln, Switzerland; 4 Department of Cardiology, Angiology, Intensive Care, cNEP, Cardiac Neuro- & Electrophysiology Research Consortium, EVK Düsseldorf, Düsseldorf, Germany; 5 DZHK (German Center for Cardiovascular Research), Partner Site Hamburg/Kiel/Lübeck, Hamburg, Germany; 6 Institute of Neural and Sensory Physiology, Heinrich Heine University Düsseldorf, Düsseldorf, Germany; 7 Department for Internal Medicine 2 – Cardiology, Angiology, and Intensive Care, Akademisches Lehrkrankenhaus, Ordensklinikum Linz Elisabethinen, Linz, Austria; 8 Klinik Für Elektrophysiologie/Rhythmologie, Herz- Und Diabeteszentrum Nordrhein-Westfalen, Universitätsklinik Der Ruhr-Universität Bochum, Bad Oeynhausen, Germany; 9 St. Joseph‘s Heart Rhythm Center, Medical College, University of Rzeszów, Rzeszów, Poland; 10 University Heart Center, Department of Cardiology, University Hospital Zurich, and University of Zurich, Zurich, Switzerland; 11 Abteilung Für Kardiologie, Klinik Floridsdorf Wien, Vienna, Austria; 12 Department of Cardiology, AZ Delta, Roeselare, Belgium; 13 Kardiologie, Frankfurt Rotkreuz Kliniken, Frankfurt am Main, Germany; 14 School of Medicine-Royal Perth Hospital Unit, University of Western Australia, Perth, Australia; 15 Department of Cardiology, Shanghai General Hospital, Shanghai Jiao Tong University School of Medicine, Shanghai, China; 16 Cardioangiologisches Centrum Bethanien (CCB), Kardiologie, Medizinische Klinik III, Agaplesion Markus Krankenhaus, Akademisches Lehrkrankenhaus der Goethe-Universität Frankfurt am Main, Frankfurt am Main, Germany

**Keywords:** Sodium-glucose co-transporter 2 inhibitors(SGLT2i), Arrhythmia, Atrial fibrillation, Atrial flutter, Ventricular tachycardia, Ventricular arrhythmia, Sudden cardiac death

## Abstract

**Objective:**

We aimed to assess the effect of SGLT2i on arrhythmias by conducting a meta-analysis using data from randomized controlled trials(RCTs).

**Background:**

Sodium-glucose co-transporter 2 inhibitors (SGLT2i) have shown cardioprotective effects via multiple mechanisms that may also contribute to decrease arrhythmias risk.

**Methods:**

We searched in databases (PubMed, Embase, Cochrane Library, and clinicaltrials.gov) up to April 2023. RCTs comparing SGLT2i with placebo were included. The effects of SGLT2i on atrial fibrillation(AF), atrial flutter(AFL), composite AF/AFL, ventricular fibrillation(VF), ventricular tachycardia(VT), ventricular extrasystoles(VES), sudden cardiac death(SCD) and composite VF/VT/SCD were evaluated.

**Results:**

33 placebo-controlled RCTs were included, comprising 88,098 patients (48,585 in SGLT2i vs. 39,513 in placebo). The mean age was 64.9 ± 9.4 years, 63.0% were male. The mean follow-up was 1.4 ± 1.1 years. The pooled-results showed that SGLT2i was associated with a significantly lower risk of AF [risk ratio(RR): 0.88, 95% confidence interval(CI) 0.78–1.00, P = 0.04] and composite AF/AFL (RR: 0.86, 95%CI 0.77–0.96, P = 0.01). This favorable effect appeared to be substantially pronounced in patients with HFrEF, male gender, dapagliflozin, and > 1 year follow-up. For SCD, only in heart failure patients, SGLT2i were found to be associated with a borderline lower risk of SCD (RR: 0.67, P = 0.05). No significant effects of SGLT2i on other ventricular arrhythmic outcomes were found.

**Conclusions:**

SGLT2i lowers the risks of AF and AF/AFL, and this favorable effect appeared to be particularly pronounced in patients with HFrEF, male gender, dapagliflozin, and longer follow-up (> 1 year). SGLT2i lowers the risk of SCD only in heart failure patients.

**Graphical Abstract:**

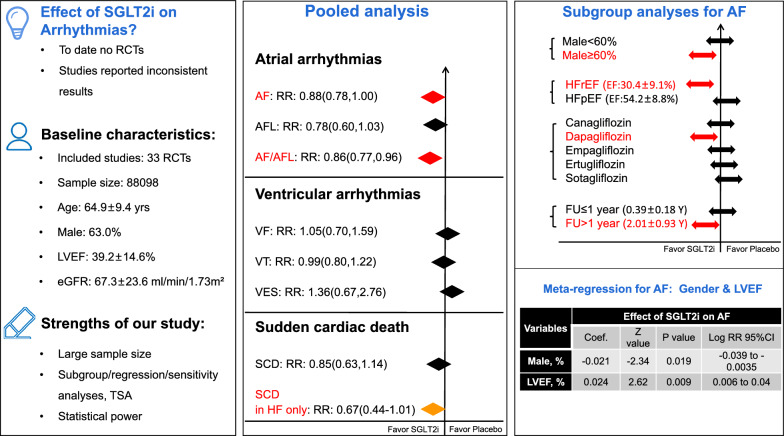

**Supplementary Information:**

The online version contains supplementary material available at 10.1186/s12933-024-02137-x.

## Introduction

Cardiac arrhythmias are associated with increased morbidity and mortality, especially in patients with Type 2 diabetes mellitus (T2DM), heart failure (HF) or chronic kidney disease (CKD) [1, 2]. Compared with non-T2DM patients, T2DM patients had a 49% and 102% increased risk of atrial fibrillation (AF) and sudden cardiac death(SCD) [3, 4]. Similarly, the prevalence of AF in patients with HF increases in parallel with the severity of HF, ranging from 10 to 26% among patients with moderate HF, and up to 50% in patients with severe HF [5]. Patients with HF carry a 6- to ninefold increased risk of SCD, around one third of all cardiac deaths are attributable to SCD [6, 7].

Sodium-glucose cotransporter 2 inhibitor (SGLT2i), initially developed as a new glucose-lowering drug, has shown a spectrum of favorable cardiovascular effects. In the latest HF guidelines from European Society of Cardiology (ESC), SGLT2i has been recommended as one standard treatment for patients with heart failure with reduced or preserved ejection fraction (HFrEF) [8]. Previous studies have shown that SGLT2i may improve myocardial energy metabolism [9, 10], suppress inflammation [11], reduce oxidative stress [12], and prevent adverse cardiac remodeling [13, 14]. Those mechanisms can also be shared in the prevention of cardiac arrhythmias, which infers that SGLT2i may be able to reduce the risk of arrhythmias.

However, there have been no randomized trials investigating the role of SGLT2i in preventing arrhythmias as a single primary endpoint. Previous retrospective studies and meta-analysis reported inconsistent results about the effects of SGLT2i on arrhythmias [15–22]. In this article we performed an updated meta-analysis using data from the most recent randomized controlled trials (RCTs) to systematically assess the effect of SGLT2i on arrhythmia events.

## Methods and materials

### Study selection and inclusion criteria

This meta-analysis was performed in accordance with the Preferred Reporting Items for Systematic Reviews and Meta-Analyses (PRISMA) guidelines. The PRISMA checklist is shown in Supplement 1. Two authors independently search in databases of PubMed, Embase, Cochrane Library, and clinical trials.gov up to April 2023. Any discrepancy between the authors was solved by an independent third author (S.C). The search terms (Sodium-glucose co-transporter 2 inhibitors or SGLT2 inhibitors or SGLT2i or canagliflozin or dapagliflozin or ertugliflozin or sotagliflozin or empagliflozin) and (diabetes mellitus or heart failure or chronic kidney disease) and (randomized controlled trial or RCT) were used with no language restrictions. The search strategy is shown in Additional file 1.

The inclusion criteria of the clinical trials: (1) RCT, (2) SGLT2i compared with placebo, (3) patients with T2DM or HF or CKD, (4) available outcome data of cardiac arrhythmias (Table 1).

**Table 1:** Pooled baseline characteristics

	Total	Randomized to SGLT2i group	Randomized to control
Sample Size, n	88,098	48,585	39,513
Age, year	64.9 ± 9.4	64.6 ± 9.4	65.2 ± 9.4
Male, n (%)	55,467/88098 (63.0)	30,318/48585 (62.4)	25,149/39513 (63.6)
LVEF, %	39.2 ± 14.6	39.3 ± 14.5	39.1 ± 14.7
eGFR, ml/min/1.73 m^2^	67.3 ± 23.6	67.9 ± 23.5	66.6 ± 23.6
HF, n (%)	27,677/80183 (34.5)	14,327/43370 (33.0)	13,350/36813 (36.3)
DM, n (%)	78,212/88098 (88.8)	43,643/48585 (89.8)	34,569/39513 (87.5)
CKD, n (%)	14,851/17851 (83.2)	7614/9107 (83.6)	7237/8744 (82.8)
AF, n (%)	6855/16045 (42.7)	3441/8024 (42.9)	3414/8021 (42.6)
ARNi use n (%)	1975/27851 (7.1)	989/13924 (7.1)	986/13927 (7.1)

*LVEF* left ventricular ejection fraction, *eGFR* estimated glomerular filtration rate, *HF* heart failure, *DM* diabetes mellitus, *CKD* chronic kidney disease, *AF* atrial fibrillation, *ARNi* angiotensin receptor/neprilysin inhibitor

### Study outcomes

Study outcomes of interest include: AF, atrial flutter (AFL), the composite events of AF/AFL, ventricular fibrillation (VF), ventricular tachycardia (VT), ventricular extrasystoles(VES), SCD and the composite events of VF/VT/SCD. The definitions of the study outcomes were derived from the original individual RCTs. The assessment of the arrhythmias was based upon routine ECGs and/or Holters scheduled both for the treatment group and the control group during the follow-up period.

The subgroup analyses were performed in patients with diabetes (ALL/NOT ALL), different types of HF (HFrEF/HFpEF), different renal function(60 ≤ eGFR < 90, 45 ≤ eGFR < 60, 30 ≤ eGFR < 45), different medications (Canagliflozin/Dapagliflozin/Empagliflozin/Ertugliflozin/Sotagliflozin) and different follow-up duration(≤ 1 year or > 1 year).

### Data collection and quality assessment

The following information was extracted from individual RCTs: (i) characteristics of studies, NCT identifier number, sample size, study design, follow-up duration; (ii) baseline characteristics of study population: age, sex, left ventricular ejection fraction(LVEF), estimated glomerular filtration rate (eGFR), comorbidities (DM, CKD, HF, AF); (iii) interventions, comparisons, and outcomes. According to the recommendations from the Cochrane Handbook for Systematic Reviews of Interventions, study quality was assessed through the Cochrane tool for assessing risk of bias (performed by J.L. and Y.C.) and disagreements were solved by consensus with a third author (S.C).

### Statistical analysis

Heterogeneity was tested using I^2^ assessed by Q test. I^2^ ≤ 50% represents low heterogeneity, a fixed-effect model with inverse variance weights was used to estimate the relative ratios (RRs) and 95% confidence interval (CI). I^2^ > 50% represents high heterogeneity, a random-effects REML model was used for RRs and 95% CI. Subgroup analysis was performed according to the baseline conditions (DM, HF, CKD), type of HF, renal function, different drug types and follow-up duration.

Fixed-effects meta-regression was performed to explore the impact of baseline characteristics on the association of SGLT2i and study outcomes. Publication bias for each endpoint was assessed by using the Egger’s test and funnel plots. We also conducted sensitivity analysis for each endpoint by excluding studies with a high/unclear overall risk of bias or studies with small sample size (n < 1000) or using odds ratio(OR) as an effect measure. To avoid potential false inferences from repetitive significance testing and underpowered meta-analysis, we performed TSA for each outcome of interest. We calculated the optimal sample size to maintain a two-sided type-I error at 0.05, a type-II errors at 0.05 or 0.20 (95% or 80% power) and the relative risk reduction (RRR) at 0.2 or 0.3, respectively.

A 2-sided P < 0.05 was considered statistically significant for analyses. Statistical analyses and TSA were performed using the Stata Software (version 16) and TSA (version 0.9).

## Results

### Baseline characteristics of included studies

A total of 7938 records were identified, of which 33 placebo-controlled RCTs met the inclusion criteria, comprising a total sample size of 88,098 participants (48,585 in SGLT2i group and 39,513 in placebo group). The flow diagram of the literature search and the study selection for this meta-analysis is shown in Additional file 1. The quality assessment of the included RCTs was presented as a risk of bias graph and a risk of bias summary figure in Additional file 1. All RCTs were considered with high methodological quality.

As shown in Table 1, The mean age of the included patients was 64.9 ± 9.4 years and 63.0% were male. The mean follow-up duration was 1.4 ± 1.1 years. Overall, The effects of a particular type of SGLT2i were examined in six trials for canagliflozin (Additional file 2: reference 1–6), eight trials for dapagliflozin (Additional file 2: reference 7–14), fourteen trials for empagliflozin (Additional file 2: reference 15–27), three trials for ertugliflozin (Additional file 2: reference 28–30), and two trials for sotagliflozin (Additional file 2: reference 31–32). Pooled baseline characteristics (SGLT2i vs control) are presented in Supplement 6. The patients’ baseline characteristics of individual trials are provided in the Additional file 1.

### Atrial arrhythmias

**Fig. 1:**
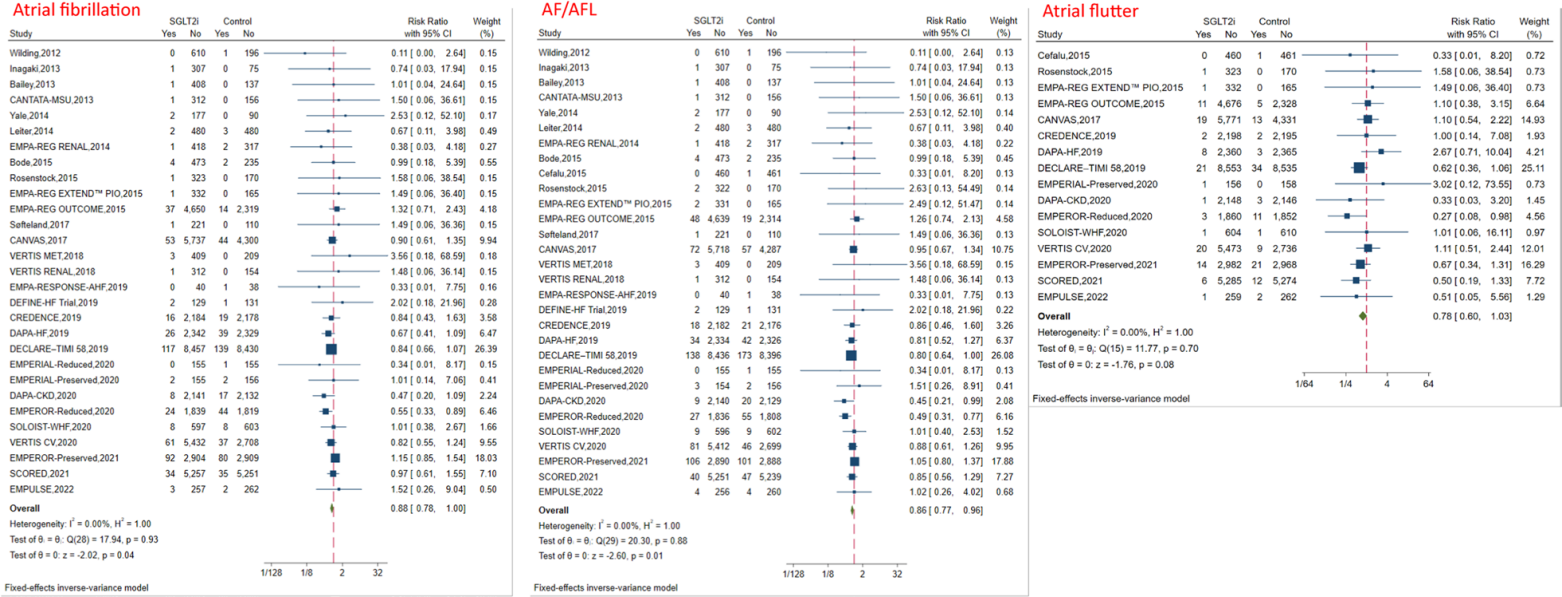
Effect of SGLT2i on atrial arrhythmias (atrial fibrillation, atrial flutter and composite events of atrial fibrillation and atrial flutter)

There were 29 RCTs that reported AF events, 16 RCTs that involved AFL events, and 30 RCTs that involved the composite events of AF/AFL. After pooling those trials, patients receiving SGLT2i were found to have a significantly lower risk of AF [risk ratio (RR): 0.88, 95% confidence interval (CI) 0.78–1.00, P = 0.04, I^2^ = 0%)] and the composite events of AF/AFL (RR: 0.86, 95%CI 0.77–0.96, P = 0.01, I^2^ = 0%). However, there was no significant association between SGLT2i and the incidence of AFL (RR: 0.78, 95%CI 0.60–1.03, P = 0.08, I^2^ = 0%) (Fig. 1).

**Fig. 2:**
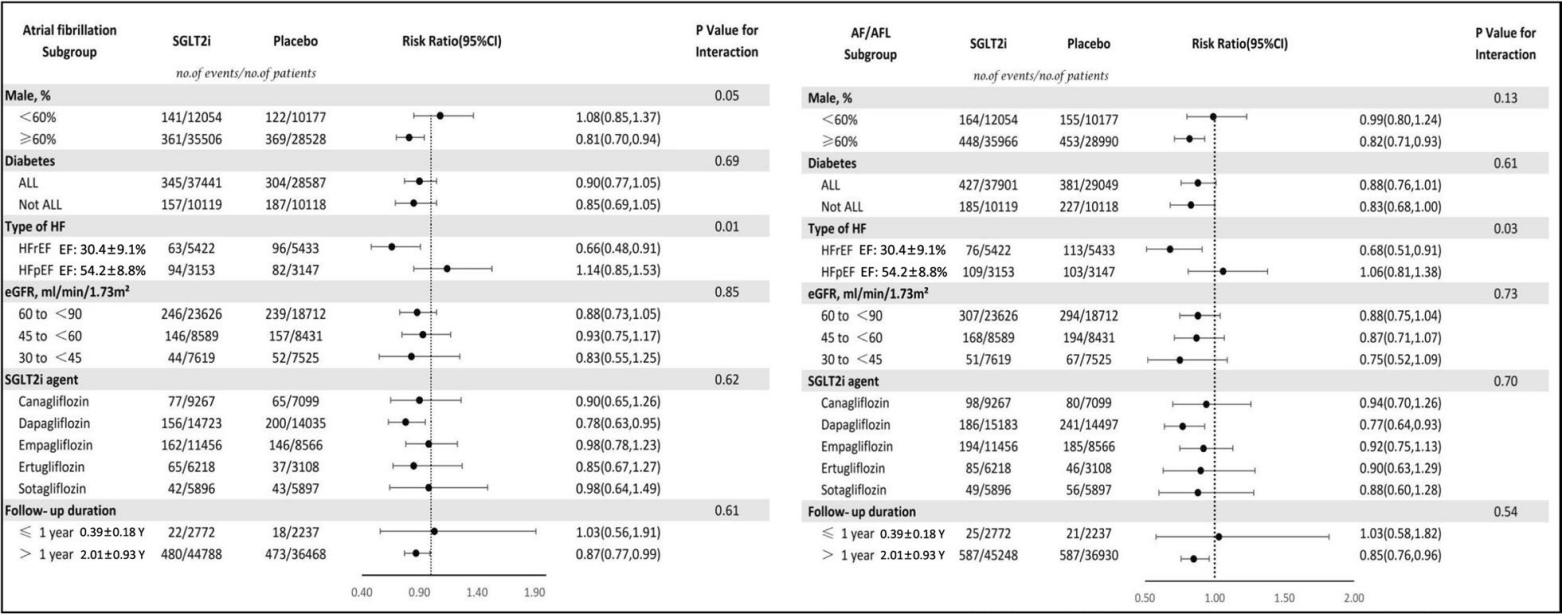
Subgroup analyses: Effect of SGLT2i on atrial fibrillation and the composite events of atrial fibrillation and atrial flutter

Subgroup analyses were then performed based on different baseline conditions. For the endpoint of AF, the favorable effect of SGLT2i appeared to be substantially pronounced in patients with HFrEF (RR: 0.66, 95%CI 0.48–0.91), Dapagliflozin (RR: 0.78, 95%CI 0.63–0.95), > 1 year follow-up (RR: 0.87, 95%CI 0.77–0.99), and studies dominantly (≥ 60%) including men (RR: 0.81, 95%CI 0.70–0.94) (Fig. 2).

**Fig. 3:**
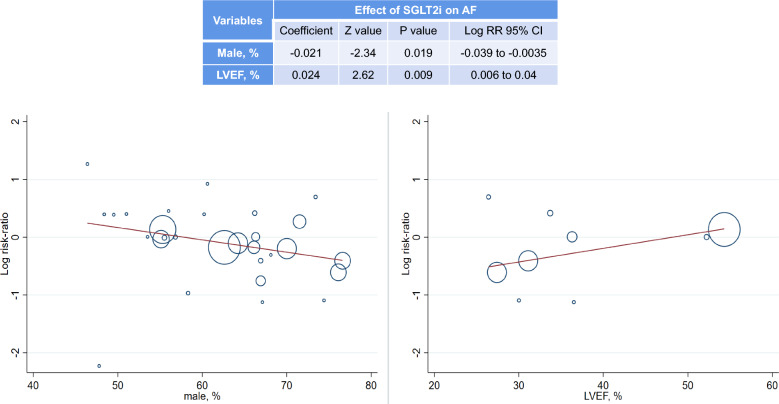
Meta-regression: Gender & LVEF correlated the effect of SGLT2i on AF

As shown in Fig. 3, further meta-regression analysis demonstrated that male gender was positively (Coefficient: − 0.021, Z value: − 2.34, Log RR 95% CI − 0.039 to − 0.0035, P: 0.019) correlated with the favorable effect of SGLT2i on AF, and LVEF was negatively (Coefficient: 0.024, Z value: 2.62, Log RR 95% CI 0.006 to 0.04, P: 0.009) correlated with the favorable effect of SGLT2i on AF.

For the composite endpoint of AF/AFL, consistently, the favorable effect of SGLT2i appeared to be substantially pronounced in patients with HFrEF (RR: 0.68, 95%CI 0.51–0.91), Dapagliflozin (RR: 0.77, 95%CI 0.64–0.93), > 1 year follow-up (RR: 0.85, 95%CI 0.76–0.96), and studies dominantly (≥ 60%) including men (RR: 0.82, 95%CI 0.71–0.93) (Fig. 2). However for the endpoint of AFL, no significant association between SGLT2i and incidence of AFL was found in the subgroup analyses Additional file 1.

### Ventricular arrhythmias and sudden cardiac death

**Fig. 4:**
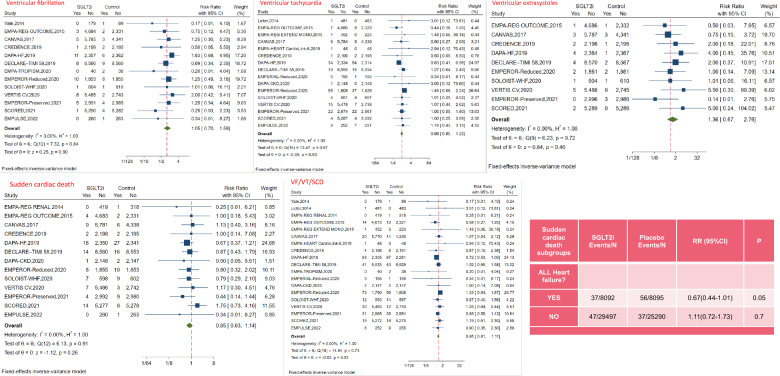
Effect of SGLT2i on ventricular arrhythmias (ventricular fibrillation, ventricular tachycardia, ventricular extrasystoles and sudden cardiac death)

There were 13 RCTs that involved VF events, 16 RCTs that involved VT events, 10 RCTs that involved VES events, 18 RCTs that involved SCD events, and 21 RCTs that involved the composite events of VF/VT/SCD. The meta-analysis indicated that there was no significant association between SGLT2i and the incidence of VF (RR: 1.05, 95%CI 0.70–1.59, P = 0.80), VT (RR: 0.99, 95%CI 0.80–1.22, P = 0.93), VES (RR: 1.36, 95%CI 0.67–2.76, P = 0.40), SCD (RR: 0.85, 95%CI 0.63–1.14, P = 0.26), and the composite events of VF/VT/SCD (RR: 0.95, 95%CI 0.81–1.11, P = 0.53) (Fig. 4).

In subgroup analysis for SCD, only heart failure patients receiving SGLT2i were found to have a borderline lower risk of SCD (RR: 0.67, 95%CI 0.44–1.01, P = 0.05) (Fig. 4). Subgroup analyses according to the baseline conditions, type of HF, renal function, different drug types and follow-up duration for ventricular arrhythmias outcomes and SCD are shown in Supplement 8.

### Sensitivity analyses and publication bias

Sensitivity analyses excluding studies with a high/unclear overall risk of bias, or excluding studies with small sample size (n < 1000), or using OR as an effect measure yielded consistent results (Additional file 1). P-value of 0.10 for Egger’s test suggested non-significant risk of publication bias. The Egger’s tests and funnel plots analysis for all outcomes did not reveal significant asymmetry (Additional file 1).

### Estimation of statistical power

Additional file 1 show estimation of statistical power [type-I error (α),type-II errors(1-β),RRR] and the sample size needed for each comparison of arrhythmia events, respectively. Analyzing the outcome of AF, the composite events of AF/AFL, VT and the composite events of VF/VT/SCD had adequate statistical power (1 − β = 80%, RRR = 0.3). While underpowered estimation was observed when analyzing AFL, VF, VES and SCD. After increasing the statistical power to 95% (1 − β = 95%, RRR = 0.2), the sample sizes remained adequate for analyzing the outcomes of AF and the composite AF/AFL. The GRADE assessment for each outcome is shown in Supplement 14. The certainty of evidence for AF, the composite events of AF/AFL, VT and the composite events of VF/VT/SCD were graded as high, whereas the certainty other outcomes were graded as moderate.

## Discussion

**Fig. 5:**
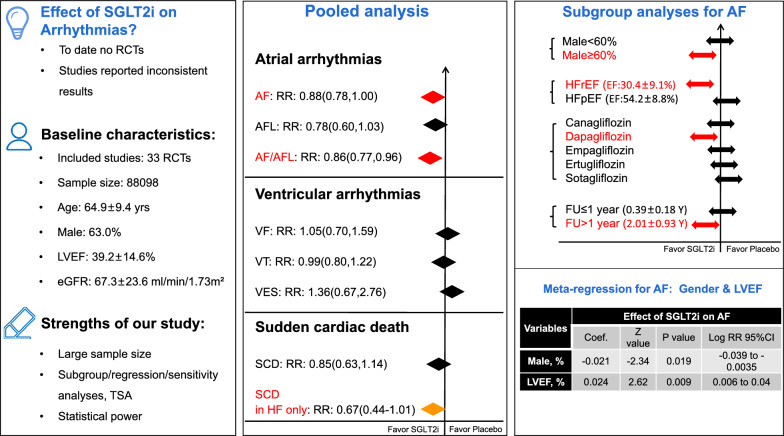
Central illustration—Insight from a large meta-analysis of randomized data focusing on effect of SGLT-2 inhibitors on arrhythmia events

As illustrated in (Fig. 5), in this large meta-analysis including 33 placebo-controlled RCTs, we found that SGLT2i is associated with significantly lower risks of AF and composite AF/AFL, specifically, the this favorable effect of SGLT2i appeared to be substantially pronounced in patients with HFrEF, male gender, dapagliflozin, and longer follow-up (> 1 year). For SCD, only in heart failure patients, SGLT2i were found to be associated with a borderline lower risk of SCD. No significant effects of SGLT2i on other outcomes were found. To our knowledge, this is up to date the largest meta-analysis that investigated the association between SGLT2i and arrhythmic events.

### The mechanisms for cardiovascular protective effects of SGLT2i

SGLT2i has been shown to be cardioprotective by multiple mechanisms that may also contribute to decrease the risk of arrhythmias. Previous studies have shown that empagliflozin can ameliorate atrial structural and electrical remodeling as well as improve mitochondrial function and mitochondrial biogenesis in T2DM, and it may be potentially used in the prevention of T2DM-related AF [23–25]. Overexpression and Ca2^+^-dependent activation of Ca2^+^/calmodulin-dependent kinase II (CaMKII) has been recognized as a key mechanism of HF, leading to contractile dysfunction and arrhythmias [26]. Mustroph et al. found that SGLT2i can reduce CaMKII activity and CaMKII-dependent sarcoplasmic reticulum Ca2^+^ leak, which may contribute to the favorable effects of SGLT2i on improving cardiac function and preventing arrhythmias [27]. In addition, SGLT2i has been found to inhibit the sodium-hydrogen exchange in myocardial cells, suppress the sympathetic nervous system and reduce the accumulation and inflammation of perivisceral adipose tissue, leading to antiarrhythmic effect [28–30].

### SGLT2i and atrial arrhythmias

We found that compared with placebo, SGLT2i was related to a 12% lower risk of AF and a 14% lower risk of the composite events of AF/AFL. The effect of SGLT2i on atrial arrhythmias has been reported in a previous meta-analysis. Li et al. identified a 18% risk reduction in both AF and the composite events of AF/AFL with SGLT2i treatment [18]. However, in the present meta-analysis, substantially more patients (88,098 vs. 52,115) and larger number of RCTs (33 vs. 22) including recently published RCTs were enrolled, which allows for further subgroup analyses, regression analysis, and statistical power estimation. Thus the results found in our analysis appeared to be robust and relevant.

Further, our subgroup analysis found that SGLT2i significantly reduced the risk for AF and composite AF/AFL by 34% and 32% in patients with HFrEF, whereas there was no significant association between SGLT2i and risk of AF or composite AF/AFL in patients with HFpEF. It’s known that AF and HFrEF frequently coexist and significantly promote each other. The mechanism underlying the association between AF and HFrEF has be recognized as multifactorial, e.g. atrial pressure overload, altered myocardial conduction, structural and electrical remodeling, autonomic nervous disorder, and maladaptive gene expression etc. It has been reported that development of AF is linked with a significantly increased risk of HF re-hospitalization, all-cause mortality and stroke in patients with HFrEF [31]. Therefore, reducing the risk of AF represents an important target in patients with HF. As for HFpEF, our meta-analysis did not detect a significant association between SGLT2i therapy and lowed risk of atrial arrhythmias. One potential explanation may be that there were only two RCTs (EMPERIAL-Preserved trial and EMPEROR-Preserved trial) including patients with HFpEF. The DELIVER-HFpEF trial (NCT03619213) also explored the effects of SGLT2i in patients with HFpEF, but the arrhythmias-related results have not been published. Therefore further studies are needed before reaching definitive conclusion.

Interestingly, as shown in Fig. 4, we found that male gender was positively correlated with the favorable effect of SGLT2i on AF, suggesting that SGLT2i reduces the risk of AF more significant in men than it does in women. This result appears to be in line with the finding from a previous meta-analysis which showed that the reduction in MACE events with SGLT2i was significantly greater in men with diabetes than that in women [32]. It should be noted that in all the enrolled RCTs, male patients were mainly included, and 63% patients in this meta-analysis were male. This indicated the underrepresentation of female inclusion in SGLT2i trials, and further studies focusing on gender difference of SGLT2i in terms of different outcomes may be needed.

Moreover, different follow-up duration may impact the effect of SGLT2i. We found in the subgroup analyses that, pooled-results from RCTs with follow-up duration > 1 year yielded significant decreased risk of AF and composite AF/AFL (RR: 0.87, 95%CI 0.77–0.99 for AF; RR: 0.85, 95%CI 0.76–0.96 for the composite events of AF/AFL) in patients treated with SGLT2i, whereas there was no significant association found between SGLT2i and lowed risk of atrial arrhythmias in trials with < 1 year follow-up. This may suggest that, a shorter follow-up duration may be insufficient for SGLT2i to achieve significant effect on AF reduction.

In addition, our meta-analysis found that dapagliflozin was the only SGLT2i agent to substantially decrease the risk of AF or AF/AFL, while other SGLT2i agents showed only neutral effect on these endpoints. This raises the hypothesis that the antiarrhythmic properties of SGLT2i might be drug specific rather than class related, nonetheless, further researches remain warranted.

### SGLT2i and ventricular arrhythmias

SGLT2i has shown to reduce all-cause and cardiovascular mortality in patients regardless of the presence of T2DM, HF, or CKD, however the association between SGLT2i and ventricular arrhythmias has been less well studied. In this meta-analysis we found that there were no significant differences in VT, VF, VES, and the composite events of VF/VT/SCD between the SGLT2i and placebo groups in overall comparisons or in subgroup analyses. However, we found that only in heart failure patients, SGLT2i were found to be associated with a borderline lower risk of SCD. Our finding seemed to be consistent with the results from an earlier meta-analysis [19]. In the DAPA-HF trial, SGLT2i resulted in lower incidents of ventricular arrhythmias (VT, VF, torsade de pointes), resuscitated cardiac arrest and SCD (5.9% vs 7.4%, HR: 0.79, P = 0.037) [33]. It should be noted that in the DAPA-HF trial, all patients had HF with reduced EF (31 ± 7%) and 55% of the patients had known ischemic cardiomyopathy.

What’s clinically relevant is that, our study found that SGLT2i lowered the risk of SCD only in heart failure patients instead of patients without HF, it’s therefore extrapolated that the effect of SGLT2i on reducing the SCD (or malignant ventricular arrhythmias) may be greater in those who had higher cardiovascular risk. On the other hand, our TSA and for sample size analysis suggested that the statistical power in terms of VF, VES and SCD was not adequate, thus further studies are needed to evaluate the association between SGLT2i and ventricular arrhythmias or SCD.

### SGLT-2i: more HF treatment than the antiarrhythmic effect?

As discussed, SGLT2i improves the prognosis of HF mainly by improving myocardial energy metabolism, suppressing inflammation, reducing oxidative stress, and preventing adverse cardiac remodeling, etc. These mechanisms can also be shared pathways in the prevention of cardiac arrhythmias. Currently, there is limited number of (preclinical or clinical) studies particularly focusing on the antiarrhythmic effect of the SGLTi. Based on the findings of our meta-analysis, the pronounced favorable effect of SGLT-2i in reducing the risk of arrhythmia was dominantly seen in patients with HFrEF rather than those without HF, it’s therefore likely that the reduced rate of arrhythmia from SGLTi seemed to be attributed to HF treatment instead of direct antiarrhythmic effect.

### Strengths and limitations

Different from other studies, our study included the most recent RCTs, consisted of large sample size and wide spectrum of patient population, by which it increased the statistical power and enabled comprehensive, clinically-relevant subgroup analyses. We also looked into a broad spectrum of arrhythmias and events as our study outcomes, including AF, AFL, VF, VT, VES, SCD. All the included RCTs had high-quality in methodology, and no significant publication bias was observed. The robustness and stability of our primary results were further tested by sensitivity analysis, TSA and sample size estimation. Furthermore, meta-regression analyses and comprehensive subgroup analyses were performed based on gender percentage, diabetes status, HFrEF/HFpEF condition, eGFR classification, different SGLT2i agents and follow-up durations, from which new, clinically relevant results were found.

Several limitations should be acknowledged. First, the intrinsic heterogeneity in study design among different trials existed. Different trials were specifically designated for different patient group (e.g. T2DM, HF or CKD). Second, our meta-analyses were based on study-level data rather than patient-level data, and unknown baseline characteristics may potentially impact the study outcomes. Although the present analysis focused on new-onset arrhythmia events, patient-level data on pre-existing arrhythmia and the use of anti-arrhythmic medications were not available from all the included trials. Third, the arrhythmia events were mainly reported as SAEs rather than primary outcome in the individual trials, and these arrhythmia outcomes were identified during follow-up visiting. Although one may argue that all the patients were randomized so that the chance of bias (if there was) should be the same in the SGLT2i and in the control group, however without continuous heart rhythm monitoring, the incidence of those arrhythmia events could have been underestimated and biased.

## Conclusion

In this large sized meta-analysis including 33 placebo-controlled RCTs, we found that SGLT2i seemed to be associated with significantly lower risks of AF and the composite events of AF/AFL, in particular, this favorable effect of SGLT2i appeared to be substantially pronounced in patients with HFrEF, male gender, dapagliflozin, and longer follow-up (> 1 year). For SCD, only in heart failure patients, SGLT2i was found to be associated with a lower risk (33% relative risk reduction) of SCD.

### Supplementary Information

**Additional file 1.** Additional tables and figures.

**Additional file 2.** Additional references.

## Data Availability

Data are available to be shared on reasonable request to the corresponding author.
