# Supplementary material for: Effect of SGLT-2 inhibitors on arrhythmia events: insight from an updated secondary analysis of > 80,000 patients (the SGLT2i—Arrhythmias and Sudden Cardiac Death)
Source: Cardiovasc Diabetol. 2024 Feb 24;23:78. doi: 10.1186/s12933-024-02137-x (PMC10893620; doi:10.1186/s12933-024-02137-x)
Supplement: Supplementary file 1 — Additional file 1. Additional tables and figures. [file 12933_2024_2137_MOESM1_ESM.pdf]

Effect of SGLT-2 inhibitors on arrhythmia events: Insight from a systematic review and pooled-analysis (the SGLT2I – Arrhythmia)

Running title: the SGLT2i - Arrhythmia

Supplementary File

- Supplement 1. PRISMA checklist
- Supplement 2. Search strategy
- Supplement 3. Flow diagram of the literature search and the study selection
- Supplement 4. Risk of bias graph of included studies
- Supplement 5. Risk of bias summary figure of included studies
- Supplement 6. Pooled baseline characteristics
- Supplement 7. The patients' baseline characteristics
- Supplement 8. The subgroup analysis of atrial flutter, ventricular fibrillation, ventricular tachycardia, ventricular extrasystoles, sudden cardiac Death and the composite events of VF/VT/SCD
- Supplement 9. Results of sensitivity analyses
- Supplement 10. The Egger's tests for all outcomes
- Supplement 11. Funnel plot analysis for all outcomes
- Supplement 12. Estimation of sample size and statistical power
- Supplement 13. Trial sequence analysis of adequate statistical powered outcomes
- Supplement 14. GRADE assessment

Supplement 1. PRISMA checklist

Section and Topic	Item #	Checklist item	Location where item is reported
**TITLE**			
Title	1	Identify the report as a systematic review.	1
**ABSTRACT**			
Abstract	2	See the PRISMA 2020 for Abstracts checklist.	2
**INTRODUCTION**			
Rationale	3	Describe the rationale for the review in the context of existing knowledge.	3
Objectives	4	Provide an explicit statement of the objective(s) or question(s) the review addresses.	3
**METHODS**			
Eligibility criteria	5	Specify the inclusion and exclusion criteria for the review and how studies were grouped for the syntheses.	3-4
Information sources	6	Specify all databases, registers, websites, organisations, reference lists and other sources searched or consulted to identify studies. Specify the date when each source was last searched or consulted.	3
Search strategy	7	Present the full search strategies for all databases, registers and websites, including any filters and limits used.	Supplement 1
Selection process	8	Specify the methods used to decide whether a study met the inclusion criteria of the review, including how many reviewers screened each record and each report retrieved, whether they worked independently, and if applicable, details of automation tools used in the process.	3
Data collection process	9	Specify the methods used to collect data from reports, including how many reviewers collected data from each report, whether they worked independently, any processes for obtaining or confirming data from study investigators, and if applicable, details of automation tools used in the process.	4
Data items	10a	List and define all outcomes for which data were sought. Specify whether all results that were compatible with each outcome domain in each study were sought (e.g. for all measures, time points, analyses), and if not, the methods used to decide which results to collect.	4
	10b	List and define all other variables for which data were sought (e.g. participant and intervention characteristics, funding sources). Describe any assumptions made about any missing or unclear information.	4
Study risk of bias assessment	11	Specify the methods used to assess risk of bias in the included studies, including details of the tool(s) used, how many reviewers assessed each study and whether they worked independently, and if applicable, details of automation tools used in the process.	4
Effect measures	12	Specify for each outcome the effect measure(s) (e.g. risk ratio, mean difference) used in the synthesis or presentation of results.	4
Synthesis methods	13a	Describe the processes used to decide which studies were eligible for each synthesis (e.g. tabulating the study intervention characteristics and comparing against the planned groups for each synthesis (item #5)).	4-5
	13b	Describe any methods required to prepare the data for presentation or synthesis, such as handling of missing summary statistics, or data conversions.	4-5
	13c	Describe any methods used to tabulate or visually display results of individual studies and syntheses.	4-5
	13d	Describe any methods used to synthesize results and provide a rationale for the choice(s). If meta-analysis was performed, describe the model(s), method(s) to identify the presence and extent of statistical heterogeneity, and software package(s) used.	4-5
	13e	Describe any methods used to explore possible causes of heterogeneity among study results (e.g. subgroup analysis, meta-regression).	5
	13f	Describe any sensitivity analyses conducted to assess robustness of the synthesized results.	5
Reporting bias assessment	14	Describe any methods used to assess risk of bias due to missing results in a synthesis (arising from reporting biases).	5
Certainty assessment	15	Describe any methods used to assess certainty (or confidence) in the body of evidence for an outcome.	5

Section and Topic	Item #	Checklist item	Location where item is reported
**RESULTS**			
Study selection	16a	Describe the results of the search and selection process, from the number of records identified in the search to the number of studies included in the review, ideally using a flow diagram.	5,Figure 1
	16b	Cite studies that might appear to meet the inclusion criteria, but which were excluded, and explain why they were excluded.	5
Study characteristics	17	Cite each included study and present its characteristics.	5,Supplement 4
Risk of bias in studies	18	Present assessments of risk of bias for each included study.	5,Supplement 2,3
Results of individual studies	19	For all outcomes, present, for each study: (a) summary statistics for each group (where appropriate) and (b) an effect estimate and its precision (e.g. confidence/credible interval), ideally using structured tables or plots.	6-7, Figure 2,5
Results of syntheses	20a	For each synthesis, briefly summarise the characteristics and risk of bias among contributing studies.	
	20b	Present results of all statistical syntheses conducted. If meta-analysis was done, present for each the summary estimate and its precision (e.g. confidence/credible interval) and measures of statistical heterogeneity. If comparing groups, describe the direction of the effect.	6-7,Figure 3, Supplement 5
	20c	Present results of all investigations of possible causes of heterogeneity among study results.	7, Supplement 7-8
	20d	Present results of all sensitivity analyses conducted to assess the robustness of the synthesized results.	7,Supplement 6
Reporting biases	21	Present assessments of risk of bias due to missing results (arising from reporting biases) for each synthesis assessed.	7
Certainty of evidence	22	Present assessments of certainty (or confidence) in the body of evidence for each outcome assessed.	7,Supplementary 9
**DISCUSSION**			
Discussion	23a	Provide a general interpretation of the results in the context of other evidence.	7-10
	23b	Discuss any limitations of the evidence included in the review.	10
	23c	Discuss any limitations of the review processes used.	10
	23d	Discuss implications of the results for practice, policy, and future research.	7-10
**OTHER INFORMATION**			
Registration and protocol	24a	Provide registration information for the review, including register name and registration number, or state that the review was not registered.	11
	24b	Indicate where the review protocol can be accessed, or state that a protocol was not prepared.	11
	24c	Describe and explain any amendments to information provided at registration or in the protocol.	11
Support	25	Describe sources of financial or non-financial support for the review, and the role of the funders or sponsors in the review.	11
Competing interests	26	Declare any competing interests of review authors.	11
Availability of data, code and other materials	27	Report which of the following are publicly available and where they can be found: template data collection forms; data extracted from included studies; data used for all analyses; analytic code; any other materials used in the review.	11

From: Page MJ, McKenzie JE, Bossuyt PM, Boutron I, Hoffmann TC, Mulrow CD, et al. The PRISMA 2020 statement: an updated guideline for reporting systematic reviews. BMJ 2021;372:n71. doi: 10.1136/bmj.n71

For more information, visit: http://www.prisma-statement.org/

Supplement 2. Search strategy

Intervention	#1	‘Sodium-Glucose Transporter 2 Inhibitors’ OR ‘Sodium-Glucose Transporter 2 Inhibitor’ OR ‘Sodium Glucose Transporter 2 Inhibitors’ OR ‘Sodium Glucose Transporter 2 Inhibitor’ OR ‘SGLT-2 Inhibitors’ OR ‘SGLT-2 Inhibitor’ OR ‘SGLT2 Inhibitors’ OR ‘SGLT2 Inhibitor’ OR ‘SGLT2is’ OR ‘SGLT2i’ OR ‘Gliflozins’
	#2	‘Canagliflozin’ OR ‘invokana’ OR ‘sulisent’ OR ‘TA7284’ OR ‘JNJ28431754’
	#3	‘Dapagliflozin’ OR ‘farxiga’ OR ‘forxiga’ OR ‘BMS512148’
	#4	‘Ertugliflozin’ OR ‘PF04971729’
	#5	‘Sotagliflozin’ OR ‘LX4221’
	#6	‘Empagliflozin’ OR ‘jardiance’ OR ‘BI10773’
	#7	#1 OR #2 OR #3 OR #4 OR #5 OR #6
Patient population	#8	‘Type 2 Diabetes Mellitus’ OR ‘Type 2 Diabetes’ OR ‘T2DM’ OR ‘T2D’ OR ‘MODY’ OR ‘NIDDM’ OR ‘Noninsulin Dependent Diabetes Mellitus’ OR ‘Maturity Onset Diabetes’ OR ‘Ketosis-Resistant Diabetes Mellitus’ OR ‘Slow-Onset Diabetes Mellitus’ OR ‘Adult-Onset Diabetes Mellitus’
	#9	‘Heart Failure’ OR ‘Cardiac Failure’ OR ‘Heart Decompensation’ OR ‘Left-Sided Heart Failure’ OR ‘Right-Sided Heart Failure’ OR ‘Myocardial Failure’ OR ‘Congestive Heart Failure’
	#10	‘Chronic Kidney Disease’ OR ‘Chronic Renal Diseases’ OR ‘Kidney Impairment’ OR ‘renal Impairment’ OR ‘Chronic Renal Insufficiency’ OR ‘Chronic Renal Insufficiencies’ OR ‘Chronic Kidney Insufficiency’ OR ‘Chronic Kidney Insufficiencies’
	#11	#8 OR #9 OR #10
Type of study	#12	‘randomized controlled trial’ OR ‘RCT’ OR ‘controlled clinical trial’ OR ‘randomized’ OR ‘placebo’
Combined	#13	#7 AND #11 AND #12

Supplement 3. Flow diagram of literature search and study selection

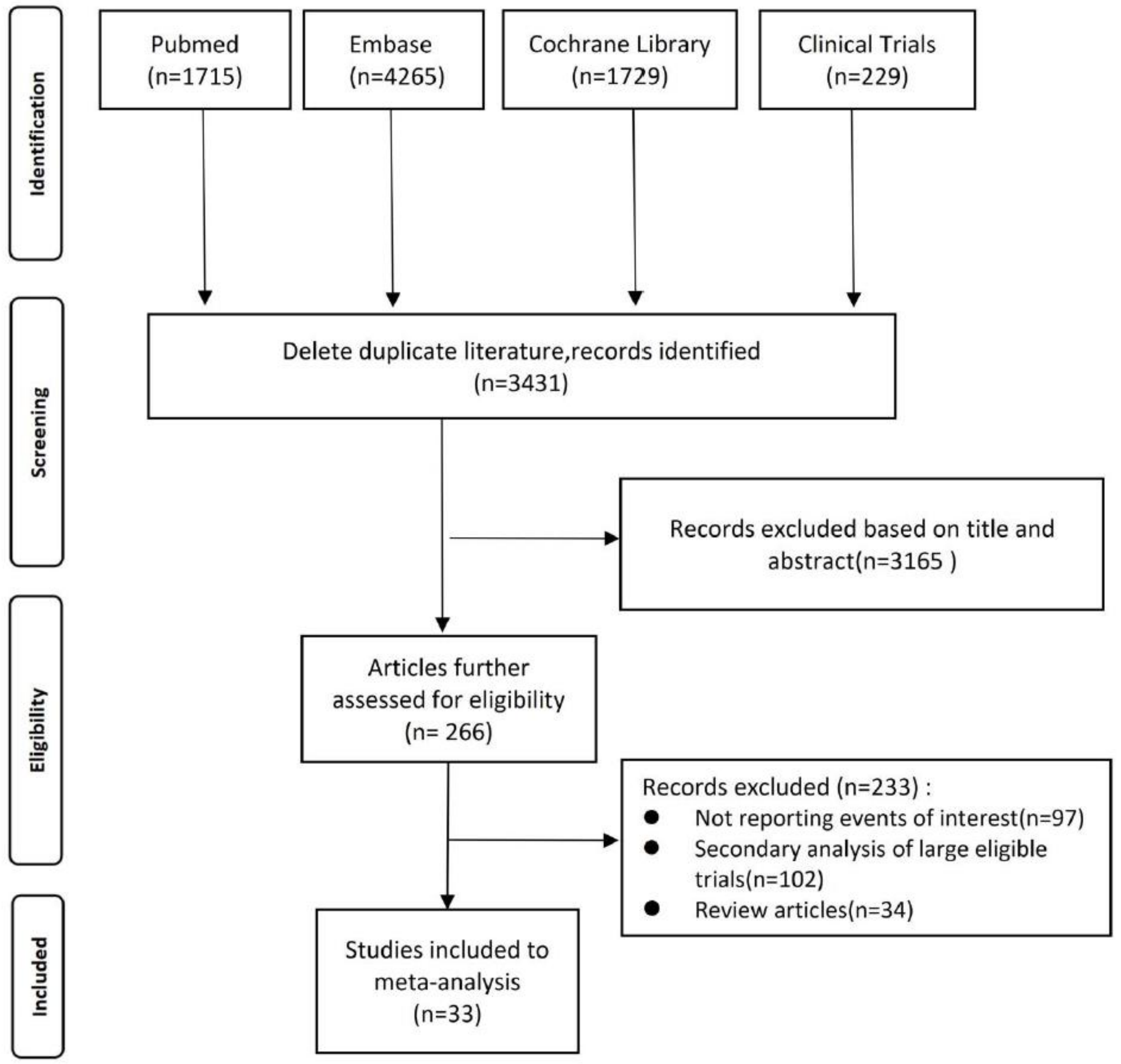

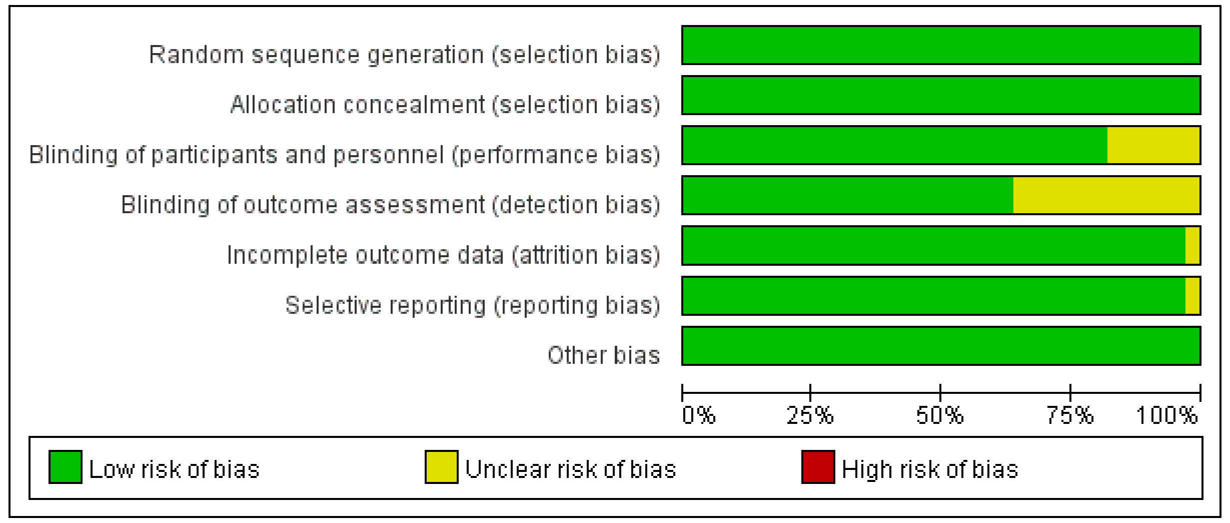

Supplement 4. Risk of bias graph of included studies

	Random sequence generation (selection bias)	Allocation concealment (selection bias)	Blinding of participants and personnel (performance bias)	Blinding of outcome assessment (detection bias)	Incomplete outcome data (attrition bias)	Selective reporting (reporting bias)	Other bias
Bailey,2013	+	+	+	+	+	+	+
Bode,2015	+	+	+	?	+	?	+
CANTATA-MSU,2013	+	+	+	+	+	+	+
CANVAS,2017	+	+	+	+	+	+	+
Cefalu,2015	+	+	?	?	+	+	+
CREDENCE,2019	+	+	+	+	+	+	+
DAPA-CKD,2020	+	+	+	+	+	+	+
DAPA-HF,2019	+	+	+	+	+	+	+
DECLARE – TIMI 58,2019	+	+	+	+	+	+	+
DEFINE-HF Trial,2019	+	+	+	+	?	+	+
EMPA-HEART CardioLink-6,2019	+	+	+	+	+	+	+
EMPA-REG EXTEND MONO,2015	+	+	+	+	+	+	+
EMPA-REG EXTEND™ PIO,2015	+	+	+	?	+	+	+
EMPA-REG OUTCOME,2015	+	+	+	+	+	+	+
EMPA-REG RENAL,2014	+	+	+	+	+	+	+
EMPA-RESPONSE-AHF,2019	+	+	+	?	+	+	+
EMPA-TROPISM,2020	+	+	+	+	+	+	+
EMPERIAL-Preserved,2020	+	+	?	?	+	+	+
EMPERIAL-Reduced,2020	+	+	?	?	+	+	+
EMPEROR-Preserved,2021	+	+	+	+	+	+	+
EMPEROR-Reduced,2020	+	+	+	+	+	+	+
EMPULSE,2022	+	+	+	?	+	+	+
Inagaki,2013	+	+	+	+	+	+	+
Leiter,2014	+	+	?	?	+	+	+
Rosenstock,2015	+	+	?	?	+	+	+
SCORED,2021	+	+	+	+	+	+	+
SOLOIST-WHF,2020	+	+	+	+	+	+	+
Søfteland,2017	+	+	+	?	+	+	+
VERTIS CV,2020	+	+	+	+	+	+	+
VERTIS MET,2018	+	+	?	?	+	+	+
VERTIS RENAL,2018	+	+	+	+	+	+	+
Wilding,2012	+	+	+	?	+	+	+
Yale,2014	+	+	+	+	+	+	+

Supplement 5. Risk of bias summary figure of included studies

Supplement 6. Pooled baseline characteristics

	Total	Randomized to SGLT2i group	Randomized to Control
Sample Size, n	88098	48585	39513
Age, y	64.9±9.4	64.6±9.4	65.2±9.4
Male, n(%)	55467/88098(63.0)	30318/48585(62.4)	25149/39513(63.6)
LVEF, %	39.2±14.6	39.3±14.5	39.1±14.7
eGFR, ml/min/1.73m²	67.3±23.6	67.9±23.5	66.6±23.6
HF, n(%)	27677/80183(34.5)	14327/43370(33.0)	13350/36813(36.3)
DM, n(%)	78212/88098(88.8)	43643/48585(89.8)	34569/39513(87.5)
CKD, n(%)	14851/17851(83.2)	7614/9107(83.6)	7237/8744(82.8)
AF history, n(%)	6855/16045(42.7)	3441/8024(42.9)	3414/8021(42.6)

LVEF: left ventricular ejection fraction, eGFR: estimated glomerular filtration rate, HF: heart failure, DM: diabetes mellitus, CKD: chronic kidney disease, AF: atrial fibrillation

Supplement 7. The patients' baseline characteristics

Study	NCT identifier number	Groups	Patients randomized (intervention/control)	Participants	Age, mean (SD), y	Male, no.(%)	LVEF,%	eGFR, ml/min/1.73m²	Patients with a history of HF,no(%)	Patients with a history of AF,no(%)	Patients with a history of DM,no(%)	Patients with a history of CKD,no(%)	Mean follow-up, y
Wilding,2012	NCT00673231	Dapagliflozin 2.5/5/10mg or Placebo	800 (607/193)	T2DM	59.3±8.2	382(47.8)	NA	NA	NA	NA	800(100)	NA	1.0
Inagaki,2013	NCT01022112	Canagliflozin 50/100/200/300mg or Placebo	382 (307/75)	T2DM	57.4±10.6	260(68.1)	NA	84.8±15.7	NA	NA	382(100)	NA	0.25
Bailey,2013	NCT00528879	Dapagliflozin 2.5mg/5mg/10mg or Placebo	546 (409/137)	T2DM	53.9±9.7	292(53.5)	NA	NA	NA	NA	546(100)	NA	2.125
CANTATA-MSU,2013	NCT01106625	Canagliflozin 100/300mg or Placbo	469 (313/156)	T2DM	56.8 ± 9.3	239 (51.0)	NA	NA	NA	NA	469(100)	NA	1.08
Yale,2014	NCT01064414	Canagliflozin 100/300mg or Placbo	269 (179/90)	T2DM with CKD	68.5±8.3	163 (60.6)	NA	39.4±6.9	NA	NA	269(100)	269(100)	1.08
Leiter,2014	NCT01042977	Dapagliflozin 10 mg or placebo	962 (480/482)	T2DM with CVD	63.7 ± 7.3	644(66.9)	NA	NA	152(15.8)	NA	962(100)	NA	0.5
EMPA-REG RENAL,2014	NCT01164501	Empagliflozin 10/25mg or Placebo	738 (419/319)	T2DM with CKD	63.9±8.8	430(58.3)	NA	NA	NA	NA	738(100)	738(100)	1.08
Bode,2015	NCT01106651	Canagliflozin 100/300mg or Placbo	714 (477/237)	T2DM	63.6 ± 6.2	396 (55.5)	NA	77.5 ± 16.6	NA	NA	714(100)	NA	2.17
Cefalu,2015	NCT01031680	Dapagliflozin10mg or Placbo	914 (455/459)	T2DM with CVD	62.9 ± 7.4	624(68.3)	NA	NA	NA	NA	914(100)	NA	1.08
Rosenstock,2015	NCT01011868	Empagliflozin 10/25mg or Placebo	494 (324/170)	T2DM	58.8 ± 9.9	276 (56)	NA	84 ± 24	NA	NA	494(100)	NA	1.625
EMPA-REG EXTEND™ PIO,2015	NCT01210001	Empagliflozin 10/25mg or Placebo	498 (333/165)	T2DM	54.5±9.8	241 (48.4)	NA	85.7±21.9	NA	NA	498(100)	NA	1.58
EMPA-REG EXTEND™ MONO ,2015	NCT01289990	Empagliflozin 10/25mg or Placebo	676 (448/228)	T2DM	54.9±11.4	410(60.7)	NA	87.3±18.4	NA	NA	676(100)	NA	1.58
EMPA-REG OUTCOME,2015	NCT01131676	Empagliflozin 10/25mg or Placebo	7020 (4687/2333)	T2DM at high CV risk	63.1±8.7	5016(71.5)	NA	74.1±21.4	706(10.1)	NA	7020(100)	NA	3.1
Søfteland,2017	NCT01734785	Empagliflozin 10/25mg or Placebo	327 (219/108)	T2DM	55.2±9.8	197(60.2)	NA	92.3±18.0	NA	NA	327(100)	NA	0.5
CANVAS,2017	NCT01989754	Canagliflozin 100/300mg or Placebo	10142 (5795/4347)	T2DM at high CV risk	63.3±8.3	6509(64.2)	NA	76.5±20.5	1461 (14.4)	NA	10142(100)	NA	3.6
VERTIS MET,2018	NCT02033889	Ertugliflozin 5/15mg or Placebo	621 (412/209)	T2DM	56.6 ± 8.8	288 (46.4)	NA	90.5 ± 19.3	NA	NA	621(100)	NA	0.54
VERTIS RENAL,2018	NCT01986855	Ertugliflozin 5/15mg or Placebo	467 (313/154)	T2DM with CKD	67.3± 8.6	231 (49.5)	NA	46.6±8.8	NA	NA	467(100)	467(100)	1.08
EMPA-HEART CardioLink-6,2019	NCT02998970	Empagliflozin 10mg or Placebo	97 (49/48)	T2DM with CAD	63.6± 10.7	90(92.8)	NA	87.8±17.6	6(6.2)	NA	97(100)	NA	0.5
EMPA-RESPONSE-AHF,2019	NCT03200860	Empagliflozin 10mg or Placebo	79 (40/39)	acute HF	75.3± 13.3	53(67.1)	36.5± 15.5	55±18	79(100)	56(70.9)	26(32.9)	NA	0.08

DEFINE-HF Trial,2019	NCT02653482	Dapagliflozin 10mg or Placebo	263 (131/132)	HF with LVEF ≤40%	61.3±11.5	193(73.4)	26.4±8.1	69.1±22.2	263(100)	106(40.3)	166(63.1)	NA	0.25
CREDENCE,2019	NCT02065791	Canagliflozin 100mg or Placebo	4401 (2202/2199)	T2DM with CKD	63.0±9.2	2907(66.1)	NA	56.2±18.2	652 (14.8)	NA	4401(100)	4401(100)	2.62
DAPA-HF,2019	NCT03036124	Dapagliflozin 10mg or Placebo	4744 (2373/2371)	HF with LVEF ≤40%	66.3±10.9	3635(76.6)	31.1±6.8	65.8±19.5	4744(100)	1818(38.3)	1983(41.8)	NA	1.5
DECLARE–TIMI 58,2019	NCT01730534	Dapagliflozin 10mg or Placebo	17160 (8582/8578)	T2DM at high CV risk	63.9±6.8	10738(62.6)	NA	85.3±15.9	1724(10.0)	NA	17160(100)	NA	4.2
EMPA-TROPISM,2020	NCT03485222	Empagliflozin 10mg or Placebo	84 (42/42)	HFrEF without DM	62±12.1	54(64)	NA	81.5±21.9	84(100)	18(21.4)	0(0)	NA	0.5
EMPERIAL-Reduced,2020	NCT03448419	Empagliflozin 10mg or Placebo	312 (156/156)	HFrEF	69.7±10.8	232(74.4)	30±7.4	57.3±22.9	312(100)	74 (23.7)	187 (59.9)	NA	0.25
EMPERIAL-Preserved,2020	NCT03448406	Empagliflozin 10mg or Placebo	315 (157/158)	HFpEF	74.0±8.9	179(56.8)	52.2±9.2	57.2±20.5	315(100)	95 (30.2)	161 (51.1)	NA	0.25
DAPA-CKD,2020	NCT03036150	Dapagliflozin 10mg or Placebo	4304 (2152/2152)	CKD	61.9±12.1	2879(66.9)	NA	43.1±12.3	468(10.9)	NA	2906(67.5)	NA	2.4
EMPEROR-Reduced,2020	NCT03057977	Empagliflozin 10mg or Placebo	3730 (1863/1867)	HF with LVEF ≤40%	66.8±11.0	2837(76.1)	27.4±6.1	61.0±21.6	3730(100)	1369(36.7)	1856(49.8)	NA	1.33
SOLOIST-WHF,2020	NCT03521934	Sotagliflozin 200/400mg or Placebo	1222 (608/614)	T2DM with worsening HF	69.7±9.3	810(66.3)	36.3±13.4	51.0±17.0	1222(100)	NA	1222(100)	NA	0.75
VERTIS CV,2020	NCT01986881	Ertugliflozin 5/15mg or Placebo	8246 (5499/2747)	T2DM with CVD	64.4±8.1	5769(70.0)	NA	NA	1958(23.7)	NA	8246(100)	NA	3.5
EMPEROR-Preserved,2021	NCT03057951	Empagliflozin 10mg or Placebo	5988 (2997/2991)	HF with LVEF >40%	71.8±9.5	3312(55.3)	54.3±8.8	60.6±19.8	5988(100)	3057(51.1)	2938(49.1)	2988(49.9)	2.18
SCORED,2021	NCT03315143	Sotagliflozin 200/400mg or Placebo	10584 (5292/5292)	T2DM with CKD and high CV risk	68.7±8.2	5830(55.1)	NA	44.3±10.7	3283(31.0)	NA	10584(100)	10584(100)	1.33
EMPULSE,2022	NCT04157751	Empagliflozin 10 mg or Placebo	530 (265/265)	HF with LVEF ≤40%	69.7±13.1	351(66.2)	33.7±18.2	52.3±22.4	530(100)	262(49.4)	240(45.3)	NA	0.25

Atrial Flutter Subgroup	SGLT2i	Placebo	Risk Ratio(95%CI)	P Value for Interaction
	no.of events/no.of patients			
**Diabetes**				0.62
ALL	82/33757	77/26882	0.82(0.59,1.13)	
Not ALL	28/9793	40/9791	0.70(0.42,1.17)	
**Heart failure**				0.89
ALL	28/8249	38/8253	0.74(0.44,1.24)	
Not ALL	69/29497	73/25290	0.77(0.55,1.09)	
**Chronic kidney disease**				0.46
ALL	8/7491	14/7483	0.57(0.24,1.38)	
Not ALL	102/36059	103/29190	0.81(0.61,1.08)	
**Type of HF**				0.85
HFrEF	13/5096	17/5106	0.79(0.35,1.78)	
HFpEF	15/3153	21/3147	0.74(0.44,1.24)	
**eGFR, ml/min/1.73m²**				0.28
60 to ＜90	61/22076	55/17949	0.90(0.62,1.31)	
45 to ＜60	22/8081	37/8082	0.61(0.36,1.05)	
30 to ＜45	7/7440	15/7435	0.47(0.19,1.15)	
**SGLT2i agent**				0.61
Canagliflozin	21/7990	15/6541	1.08(0.56,2.10)	
Dapagliflozin	30/13551	41/13548	0.72(0.44,1.17)	
Empagliflozin	32/10620	39/7942	0.69(0.42,1.13)	
Ertugliflozin	20/5493	9/2745	1.11(0.51,2.44)	
Sotagliflozin	7/5896	13/5897	0.54(0.21,1.36)	
**Follow- up duration**				0.78
≤ 1 year	3/1022	3/1033	0.98(0.20,4.73)	
＞ 1 year	107/42528	114/35640	0.78(0.60,1.03)	

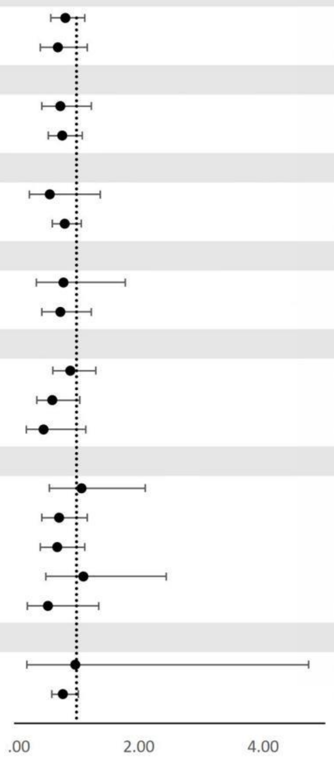

Ventricular fibrillation Subgroup	SGLT2i	Placebo	Risk Ratio(95%CI)	P Value for Interaction
	no.of events/no.of patients			
**Diabetes**				0.36
ALL	27/32819	24/26175	0.87(0.49,1.56)	
Not ALL	26/7527	21/7524	1.27(0.71,2.28)	
**Heart failure**				0.51
ALL	27/8132	22/8135	1.26(0.71,2.23)	
Not ALL	23/27348	20/23141	0.94(0.49,1.80)	
**Chronic kidney disease**				0.07
ALL	2/7670	7/7573	0.30(0.07,1.25)	
Not ALL	51/32676	38/26126	1.18(0.77,1.81)	
**Type of HF**				0.89
HFrEF	22/5096	16/5106	1.38(0.72,2.62)	
HFpEF	5/2991	4/2985	1.25(0.34,4.64)	
**eGFR, ml/min/1.73m²**				0.24
60 to ＜90	27/21459	22/17654	1.11(0.63,1.98)	
45 to ＜60	17/7924	16/7924	1.08(0.54,2.14)	
30 to ＜45	1/5470	5/5376	0.22(0.04,1.34)	
**SGLT2i agent**				0.69
Canagliflozin	6/8169	6/6631	0.78(0.25,2.46)	
Dapagliflozin	19/10942	15/10937	1.26(0.63,2.50)	
Empagliflozin	18/9846	17/7489	1.01(0.52,1.95)	
Ertugliflozin	8/5493	2/2745	2.00(0.42,9.41)	
Sotagliflozin	2/5896	5/5897	0.43(0.08,2.38)	
**Follow- up duration**				0.30
≤ 1 year	1/905	4/915	0.43(0.08,2.42)	
＞ 1 year	52/39441	41/32784	1.11(0.73,1.70)	

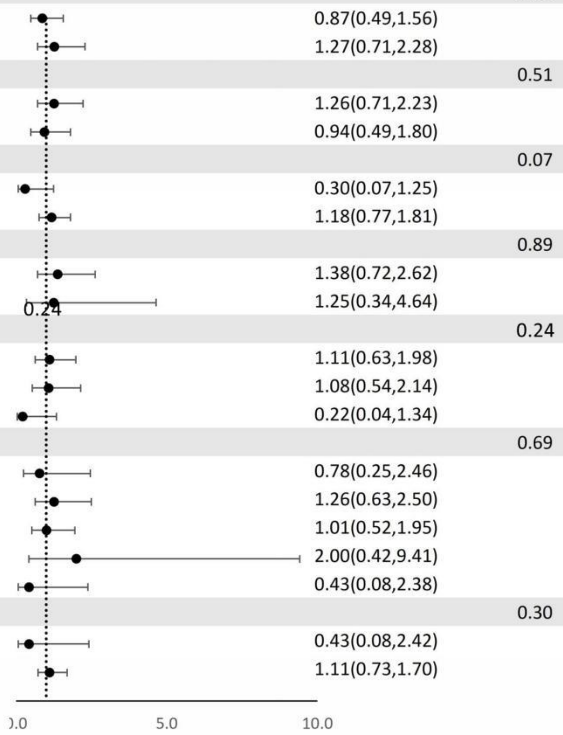

Ventricular tachycardia Subgroup	SGLT2i	Placebo	Risk Ratio(95%CI)	P Value for Interaction
	no.of events/no.of patients			
**Diabetes**				0.94
ALL	59/33624	45/26839	0.98(0.66,1.44)	
Not ALL	120/9791	121/9789	1.00(0.77,1.28)	
**Heart failure**				0.54
ALL	123/8247	125/8251	0.99(0.77,1.27)	
Not ALL	49/30481	33/26044	1.16(0.75,1.79)	
**Chronic kidney disease**				0.79
ALL	5/7491	6/7483	0.84(0.25,2.79)	
Not ALL	174/35924	160/29145	1.00(0.80,1.24)	
**Type of HF**				0.97
HFrEF	101/5251	103/5262	0.99(0.75,1.30)	
HFpEF	22/2996	22/2989	1.00(0.55,1.80)	
**eGFR, ml/min/1.73m²**				0.08
60 to ＜90	68/21921	82/17885	0.75(0.54,1.03)	
45 to ＜60	90/8079	73/8080	1.24(0.91,1.68)	
30 to ＜45	5/7440	4/7435	1.19(0.33,4.24)	
**SGLT2i agent**				0.59
Canagliflozin	7/7990	7/6541	0.80(0.28,2.32)	
Dapagliflozin	55/13573	69/13569	0.79(0.56,1.13)	
Empagliflozin	94/10463	75/7876	1.17(0.86,1.58)	
Ertugliflozin	15/5493	7/2745	1.07(0.44,2.62)	
Sotagliflozin	8/5896	8/5897	1.00(0.38,2.67)	
**Follow- up duration**				0.67
≤ 1 year	14/1551	12/1562	1.15(0.55,2.42)	
＞ 1 year	165/41864	154/35066	0.98(0.78,1.22)	

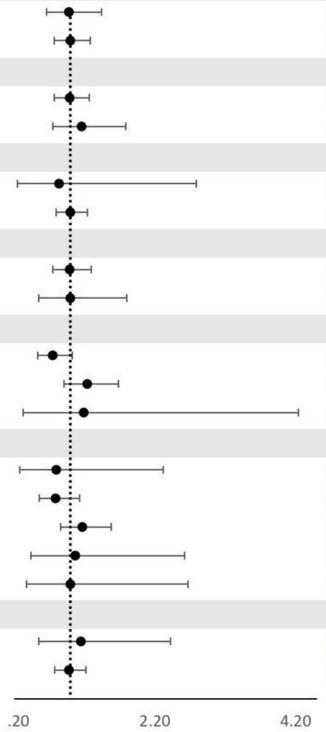

Ventricular extrasystoles Subgroup	SGLT2i	Placebo	Risk Ratio(95%CI)	P Value for Interaction
	no.of events/no.of patients			
**Diabetes**				0.46
ALL	18/32640	8/26085	1.65(0.74,3.67)	
Not ALL	6/7227	6/7220	1.00(0.34,2.96)	
**Heart failure**				0.34
ALL	7/7832	7/7831	1.00(0.36,2.75)	
Not ALL	16/27348	6/23141	1.94(0.79,4.73)	
**Chronic kidney disease**				0.38
ALL	4/7491	1/7483	3.00(0.47,19.01)	
Not ALL	20/32376	13/25822	1.23(0.62,2.46)	
**Type of HF**				0.13
HFrEF	7/4836	4/4842	1.75(0.51,5.99)	
HFpEF	0/2996	3/2989	0.14(0.01,2.76)	
**eGFR, ml/min/1.73m²**				0.41
60 to ＜90	12/21419	7/17614	1.45(0.58,3.58)	
45 to ＜60	5/7664	7/7660	0.73(0.24,2.20)	
30 to ＜45	2/5291	0/5286	5.00(0.24,104.02)	
**SGLT2i agent**				0.29
Canagliflozin	5/7990	4/6541	1.03(0.28,3.78)	
Dapagliflozin	8/10942	3/10937	2.67(0.71,10.04)	
Empagliflozin	3/9546	6/7185	0.46(0.12,1.73)	
Ertugliflozin	5/5493	0/2745	5.50(0.30,99.39)	
Sotagliflozin	3/5896	1/5897	2.34(0.35,15.89)	
**Follow- up duration**				0.83
≤ 1 year	1/605	1/611	1.01(0.06,16.11)	
＞ 1 year	23/39262	13/32694	1.38(0.66,2.89)	

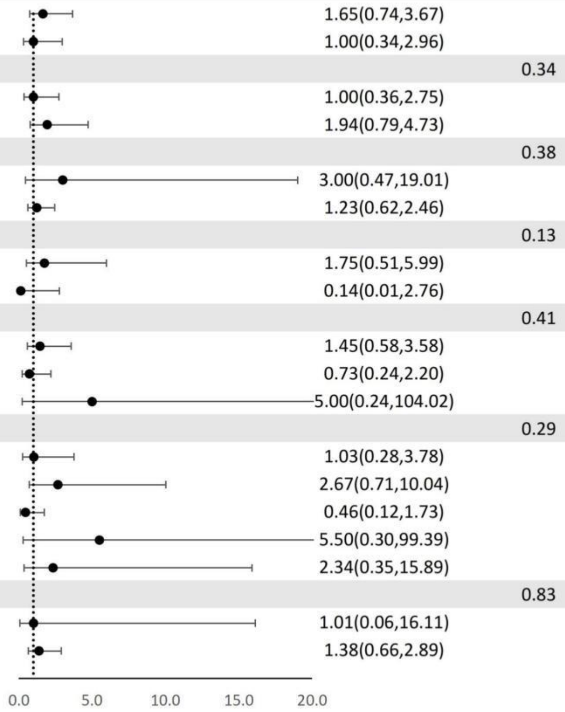

Sudden cardiac death Subgroup	SGLT2i	Placebo	Risk Ratio(95%CI)	P Value for Interaction
	no.of events/no.of patients			
**Diabetes**				0.11
ALL	57/33059	47/26404	1.05(0.71,1.55)	
Not ALL	31/9636	49/9633	0.64(0.41,1.00)	
**Heart failure**				0.10
ALL	37/8092	56/8095	0.67(0.44,1.01)	
Not ALL	47/29497	37/25290	1.11(0.72,1.73)	
**Chronic kidney disease**				0.15
ALL	16/7910	11/7802	1.43(0.66,3.10)	
Not ALL	72/34785	85/28235	0.77(0.56,1.06)	
**Type of HF**				0.46
HFrEF	33/5096	47/5106	0.71(0.46,1.10)	
HFpEF	4/2996	9/2989	0.44(0.14,1.44)	
**eGFR, ml/min/1.73m²**				0.29
60 to ＜90	45/21419	51/17614	0.81(0.54,1.21)	
45 to ＜60	21/7924	31/7924	0.69(0.40,1.20)	
30 to ＜45	15/7440	10/7435	1.51(0.67,3.42)	
**SGLT2i agent**				0.57
Canagliflozin	11/7990	8/6541	1.10(0.44,2.73)	
Dapagliflozin	33/13091	45/13086	0.73(0.47,1.15)	
Empagliflozin	16/10225	23/7768	0.64(0.34,1.21)	
Ertugliflozin	7/5493	3/2745	1.17(0.30,4.51)	
Sotagliflozin	21/5896	17/5897	1.23(0.64,2.36)	
**Follow- up duration**				0.75
≤ 1 year	7/865	10/875	0.73(0.29,1.87)	
＞ 1 year	81/41830	86/35162	0.86(0.63,1.17)	

VF/VT/SCD Subgroup	SGLT2i	Placebo	Risk Ratio(95%CI)	P Value for Interaction
	no.of events/no.of patients			
**Diabetes**				0.75
ALL	143/34222	116/27248	0.98(0.77,1.26)	
Not ALL	177/9831	191/9829	0.93(0.76,1.14)	
**Heart failure**				0.34
ALL	187/8287	203/8291	0.93(0.76,1.13)	
Not ALL	119/30481	90/26044	1.09(0.83,1.44)	
**Chronic kidney disease**				0.98
ALL	23/8089	24/7892	0.94(0.53,1.67)	
Not ALL	297/35964	283/29185	0.95(0.81,1.12)	
**Type of HF**				0.81
HFrEF	156/5251	166/5262	0.94(0.76,1.17)	
HFpEF	31/2996	35/2989	0.88(0.55,1.43)	
**eGFR, ml/min/1.73m²**				0.26
60 to ＜90	140/21961	155/17925	0.82(0.65,1.03)	
45 to ＜60	128/8079	120/8080	1.07(0.84,1.37)	
30 to ＜45	21/7619	19/7525	1.08(0.59,2.01)	
**SGLT2i agent**				0.67
Canagliflozin	24/8169	21/6631	0.91(0.50,1.64)	
Dapagliflozin	107/13569	129/13569	0.83(0.64,1.07)	
Empagliflozin	128/10922	115/8235	1.03(0.81,1.33)	
Ertugliflozin	30/5493	12/2745	1.25(0.64,2.44)	
Sotagliflozin	31/5896	30/5897	1.04(0.63,1.71)	
**Follow- up duration**				0.76
≤ 1 year	22/1591	26/1602	0.88(0.50,1.52)	
＞ 1 year	298/42462	281/35475	0.96(0.81,1.13)	

Supplement 8. The subgroup analysis of atrial flutter, ventricular fibrillation, ventricular tachycardia, ventricular extrasystoles, sudden cardiac Death and the composite events of VF/VT/SCD

Supplement 9. Results of sensitivity analyses

Outcome	Analysis	Result
Atrial fibrillation	Primary analysis	RR 0.88 (95% CI 0.78 – 1.00)
	Using OR as effect measure	RR 0.88 (95% CI 0.77 – 1.00)
	Excluding studies with high/unclear overall risk of bias	RR 0.87(95% CI 0.77 – 0.99)
	excluding studies with small sample size (n<1000)	RR 0.87(95% CI 0.77 – 0.99)
Atrial flutter	Primary analysis	RR 0.78 (95% CI 0.60–1.03)
	Using OR as effect measure	RR 0.78 (95% CI 0.60–1.03)
	Excluding studies with high/unclear overall risk of bias	RR 0.78 (95% CI 0.59–1.03)
	excluding studies with small sample size (n<1000)	RR 0.78 (95% CI 0.59–1.03)
AF/AFL	Primary analysis	RR 0.86 (95% CI 0.77-0.96)
	Using OR as effect measure	RR 0.86 (95% CI 0.76-0.96)
	Excluding studies with high/unclear overall risk of bias	RR 0.86 (95% CI 0.76-0.96)
	excluding studies with small sample size (n<1000)	RR 0.86 (95% CI 0.76-0.96)
Ventricular fibrillation	Primary analysis	RR 1.05 (95% CI 0.70-1.59)
	Using OR as effect measure	RR 1.05 (95% CI 0.70-1.59)
	Excluding studies with high/unclear overall risk of bias	RR 1.07 (95% CI 0.71-1.63)
	excluding studies with small sample size (n<1000)	RR 1.15 (95% CI 0.75-1.75)
Ventricular tachycardia	Primary analysis	RR 0.99 (95% CI 0.80-1.22)
	Using OR as effect measure	RR 0.99 (95% CI 0.80-1.23)
	Excluding studies with high/unclear overall risk of bias	RR 0.98 (95% CI 0.79-1.22)
	excluding studies with small sample size (n<1000)	RR 0.98 (95% CI 0.79-1.22)
Ventricular extrasystoles	Primary analysis	RR 1.36 (95% CI 0.67-2.76)
	Using OR as effect measure	RR 1.36 (95% CI 0.67-2.76)
	Excluding studies with high/unclear overall risk of bias	RR 1.36 (95% CI 0.67-2.76)
	excluding studies with small sample size (n<1000)	RR 1.36 (95% CI 0.67-2.76)
Sudden cardiac death	Primary analysis	RR 0.85 (95% CI 0.63-1.14)
	Using OR as effect measure	RR 0.84 (95% CI 0.63-1.14)
	Excluding studies with high/unclear overall risk of bias	RR 0.85 (95% CI 0.63-1.15)
	excluding studies with small sample size (n<1000)	RR 0.86 (95% CI 0.64-1.16)
VF/VT/SCD	Primary analysis	RR 0.95 (95% CI 0.81-1.11)
	Using OR as effect measure	RR 0.95 (95% CI 0.81-1.12)
	Excluding studies with high/unclear overall risk of bias	RR 0.95 (95% CI 0.81-1.12)
	excluding studies with small sample size (n<1000)	RR 0.96 (95% CI 0.82-1.13)

Supplement 10. The Egger's tests for all outcomes

Comparisons	Egger test	
	Z value	P value
Atrial fibrillation	-0.00	0.9966
Atrial flutter	0.41	0.6827
AF/AFL	0.16	0.8766
Ventricular fibrillation	-1.92	0.0554
Ventricular tachycardia	0.18	0.8554
Ventricular extrasystoles	0.12	0.9069
Sudden cardiac death	-0.48	0.6321
VF/VT/SCD	-0.73	0.4671

Supplement 11. Funnel plot analysis for all outcomes

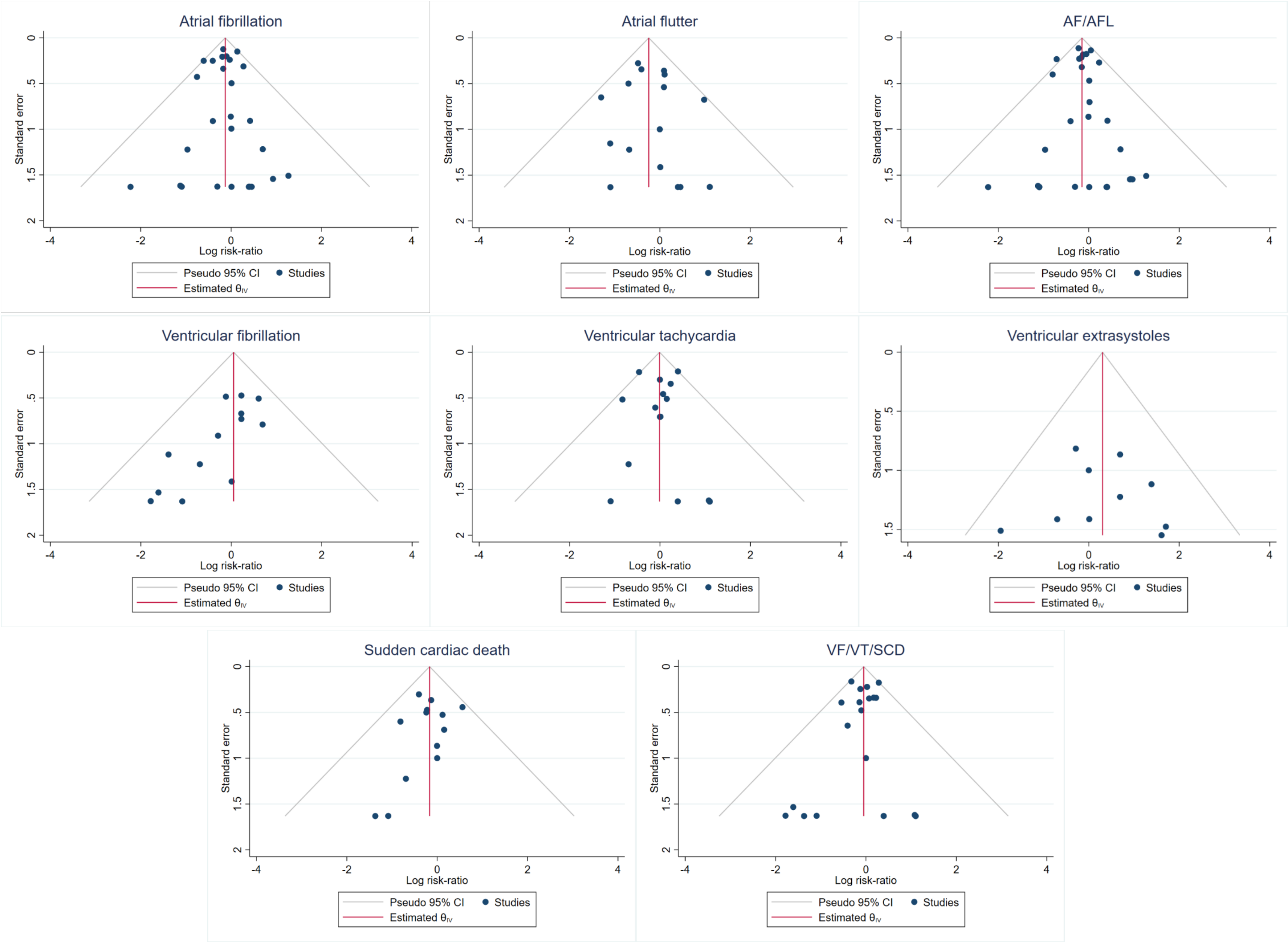

Supplement 12. Estimation of sample size and statistical power

Outcomes	Event rate in SGLT2i	Event rate in Control	Sample size actually included	α	1-β P0WER	RRR	Sample size needed	outcome reliable?
Atrial fibrillation	502/47560 1.06%	491/38705 1.27%	86265	5%	80%	0.3	23228	YES
					95%	0.2	94135	YES
Atrial flutter	110/43550 0.25%	117/36673 0.32%	80223	5%	80%	0.3	84540	NO
AF/AFL	612/48020 1.27%	608/39167 1.55%	87187	5%	80%	0.3	18444	YES
					95%	0.2	74401	YES
Ventricular fibrillation	53/40346 0.13%	45/33699 0.13%	74045	5%	80%	0.3	215607	NO
Ventricular tachycardia	179/43415 0.41%	166/36628 0.45%	80043	5%	80%	0.3	71247	YES
					95%	0.2	258842	NO
Ventricular extrasystoles	24/39867 0.06%	14/33305 0.04%	73172	5%	80%	0.3	392248	NO
Sudden cardiac death	88/42695 0.21%	96/36037 0.27%	78732	5%	80%	0.3	112569	NO
VF/VT/SCD	320/44053 0.73%	307/37077 0.83%	81130	5%	80%	0.3	35165	YES
					95%	0.2	132996	NO

Supplement 13. Trial sequence analysis of adequate statistical powered outcomes

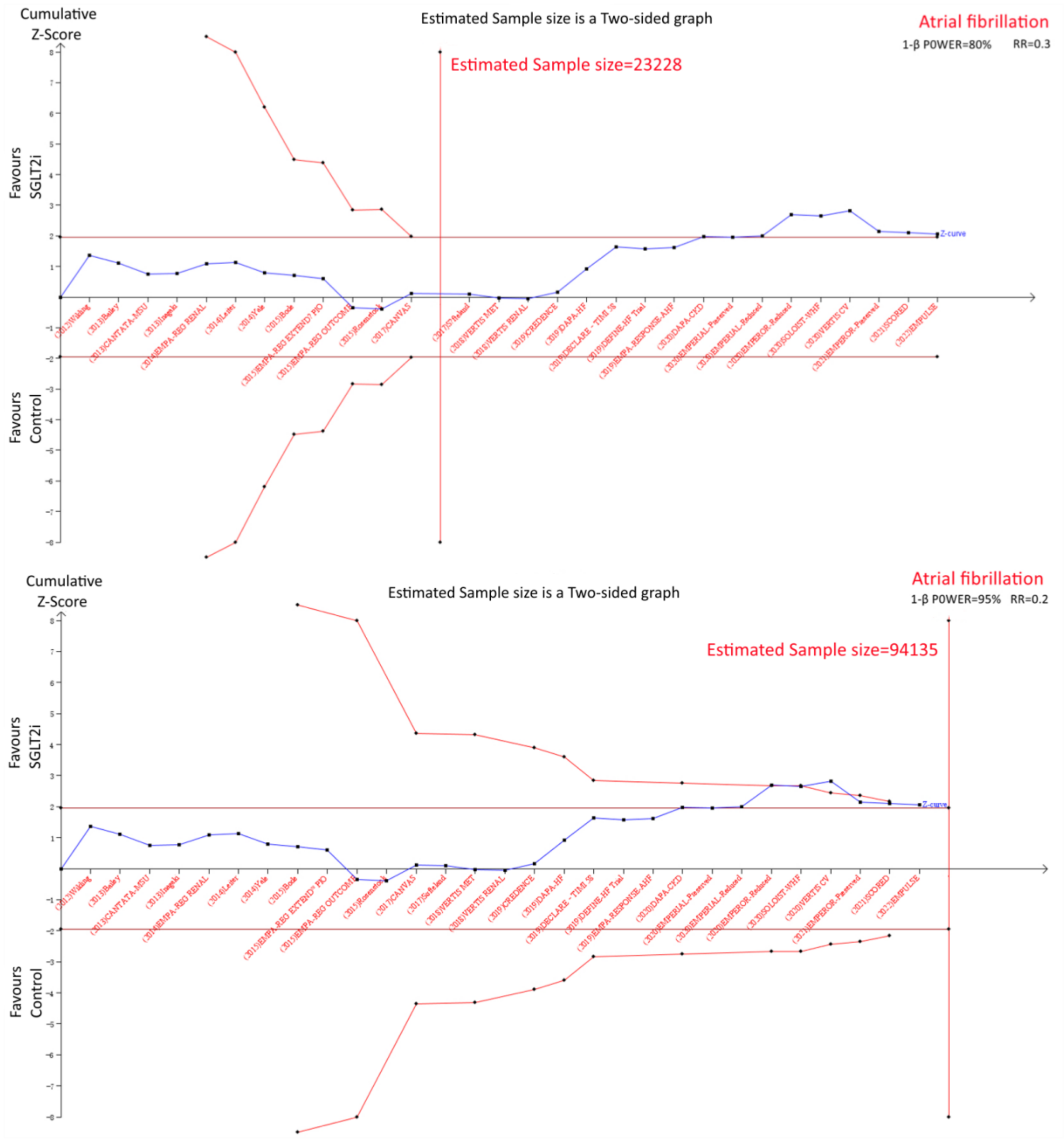

Figure (a). Trial sequence analysis for Atrial fibrillation

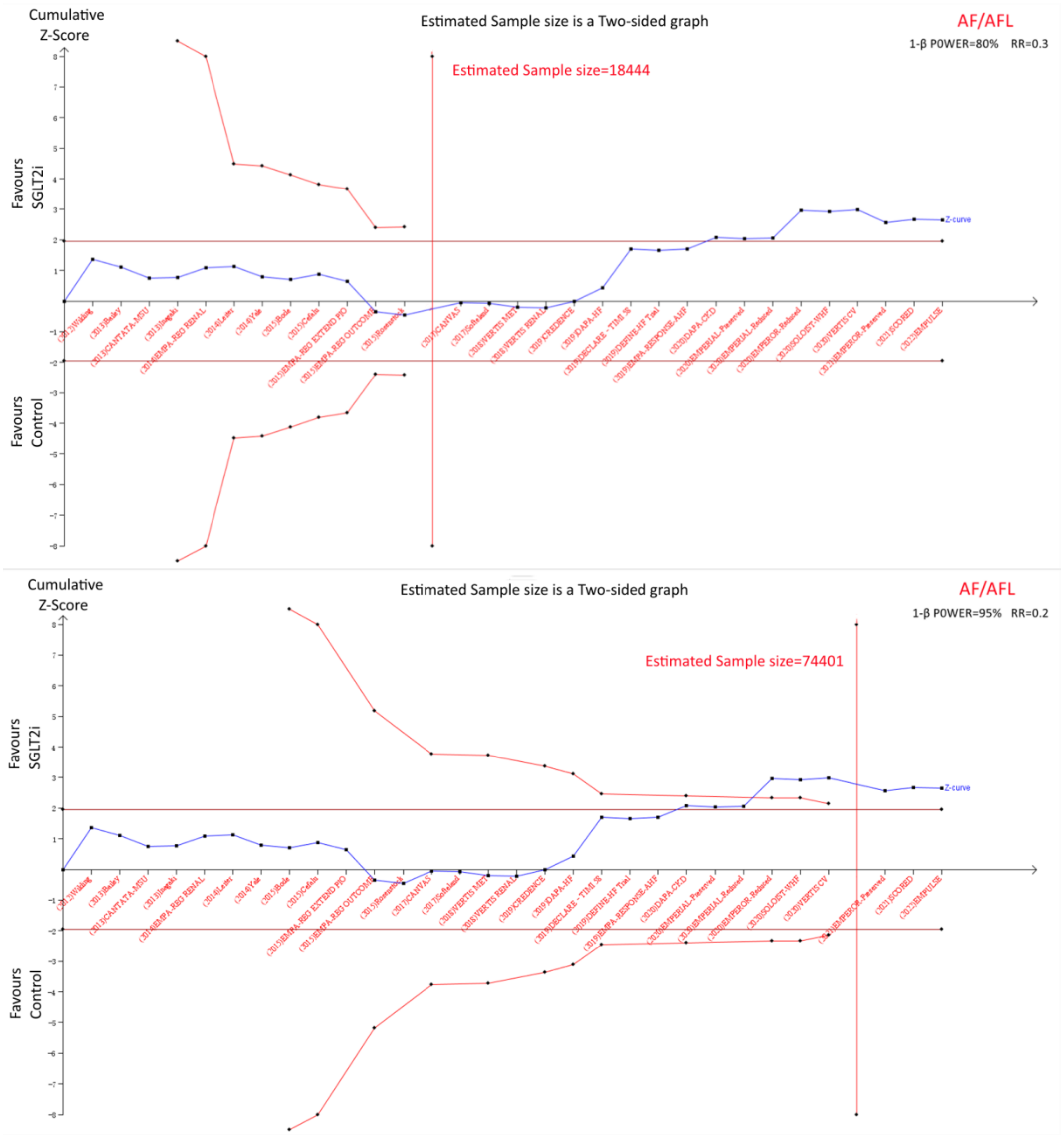

Figure (b). Trial sequence analysis for the composite AF/AFL

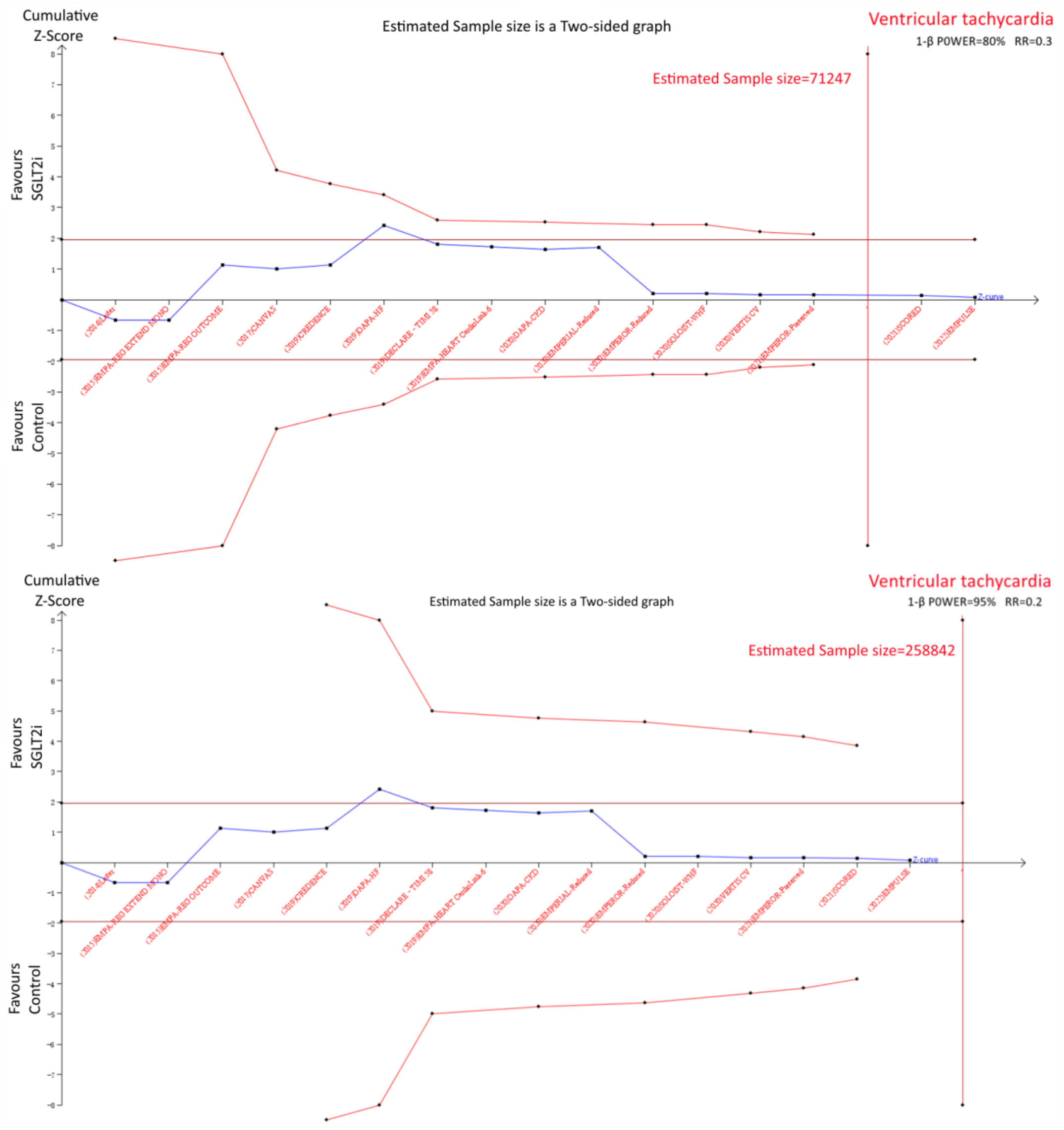

Figure (c). Trial sequence analysis for Ventricular tachycardia

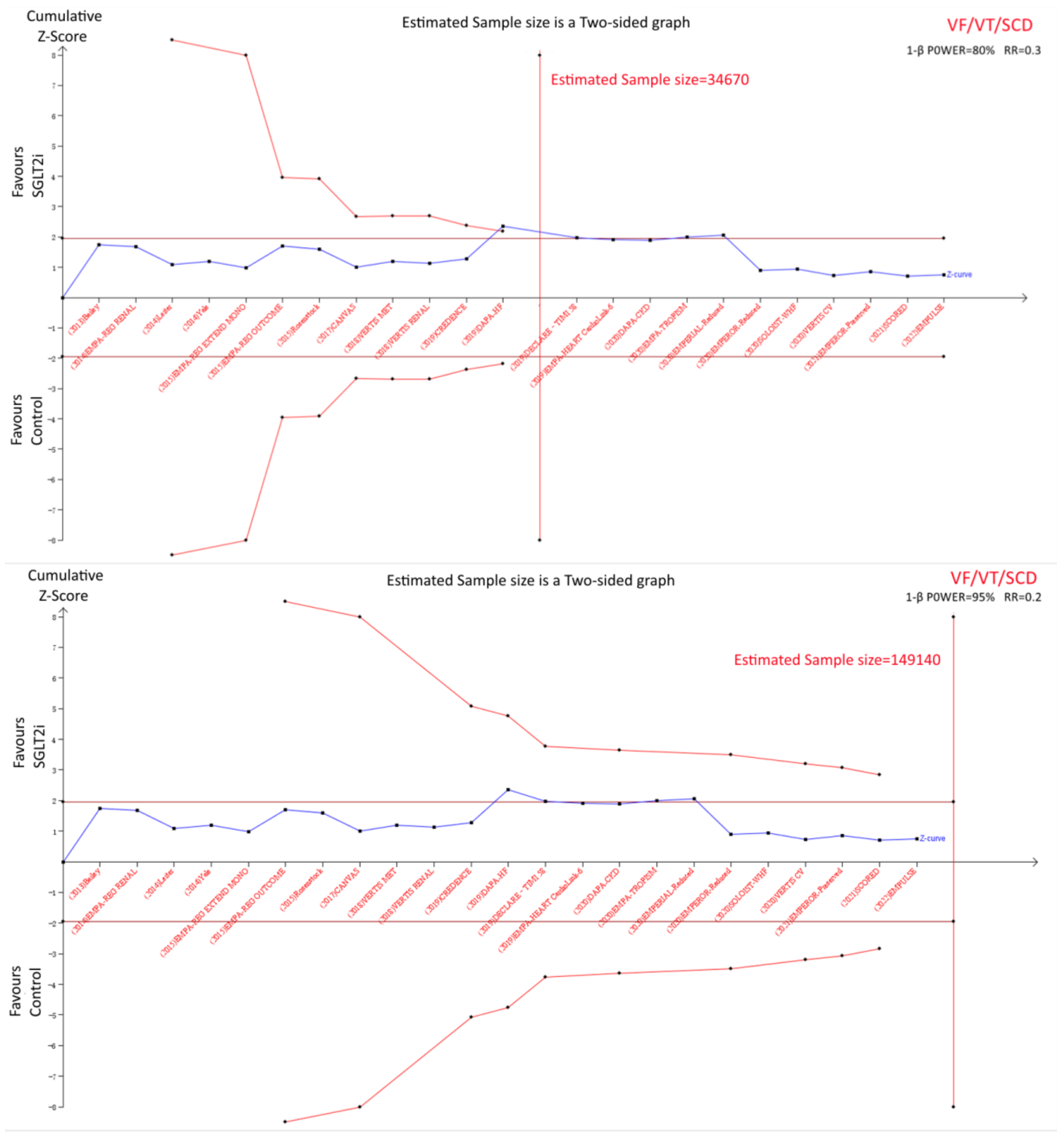

Figure (d). Trial sequence analysis for the composite VF/VT/SCD

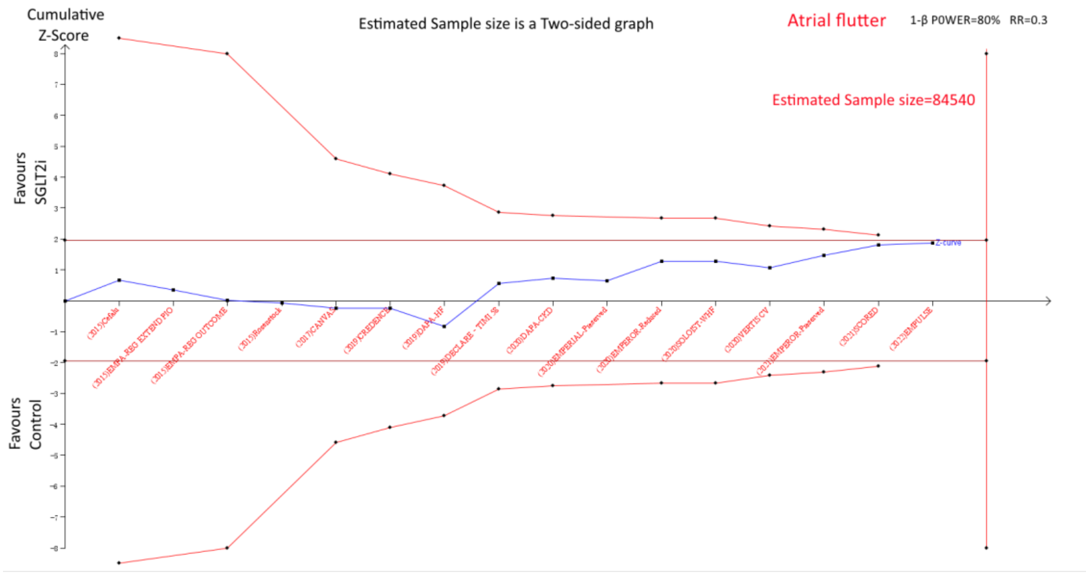

Figure (e). Trial sequence analysis for Atrial flutter

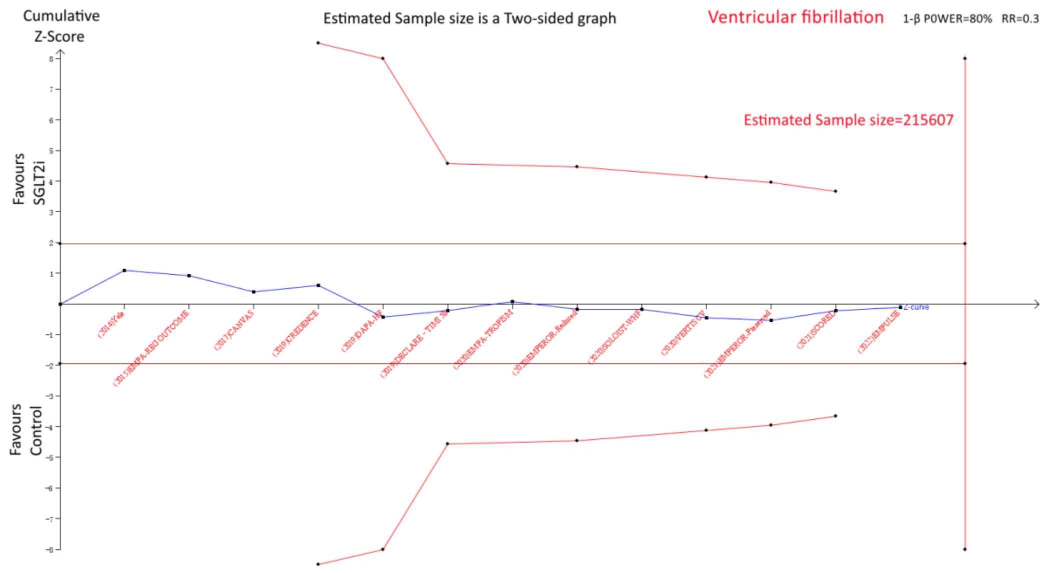

Figure (f). Trial sequence analysis for Ventricular fibrillation

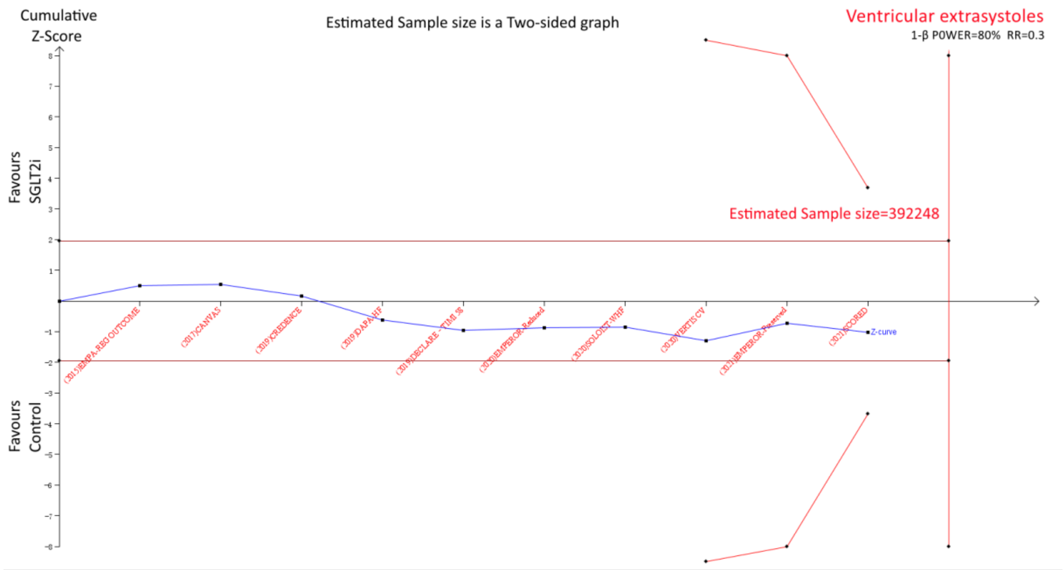

Figure (g). Trial sequence analysis for Ventricular extrasystoles

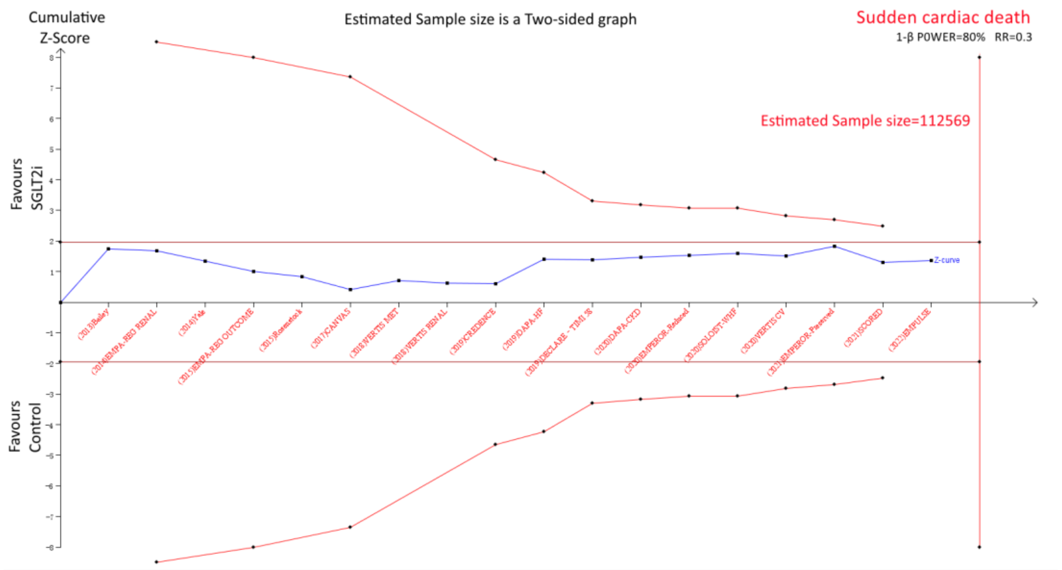

Figure (h). Trial sequence analysis for Sudden cardiac death

Supplement 14. GRADE assessment

Quality assessment							No of patients		Effect		Quality	Importance
**No of studies**	**Design**	**Risk of bias**	**Inconsistency**	**Indirectness**	**Imprecision**	**Other considerations**	**SGLT2i**	**Control**	**Relative (95% CI)**	**Absolute**		
**Atrial fibrillation**												
29	randomised trials	no serious risk of bias	no serious inconsistency	no serious indirectness	no serious imprecision	none	502/47560 (1.1%)	491/38705 (1.3%)	RR 0.88 (0.78 to 1.00)	2 fewer per 1000 (from 3 more to 0 more)	⊕⊕⊕⊕ HIGH	CRITICAL
**Atrial flutter**												
16	randomised trials	no serious risk of bias	no serious inconsistency	no serious indirectness	serious[1]	none	110/43550 (0.25%)	117/36673 (0.32%)	RR 0.78 (0.60 to 1.03)	1 fewer per 1000 (from 1 more to 0 more)	⊕⊕⊕O MODERATE	IMPORTANT
**AF/AFL**												
30	randomised trials	no serious risk of bias	no serious inconsistency	no serious indirectness	no serious imprecision	none	612/48020 (1.3%)	608/39167 (1.6%)	RR 0.86 (0.77 to 0.96)	2 fewer per 1000 (from 1 more to 4 more)	⊕⊕⊕⊕ HIGH	CRITICAL
**Ventricular fibrillation**												
13	randomised trials	no serious risk of bias	no serious inconsistency	no serious indirectness	serious[1]	none	53/40346 (0.13%)	45/33600 (0.13%)	RR 1.05 (0.70 to 1.59)	0 more per 1000 (from 0 more to 1 more)	⊕⊕⊕O MODERATE	CRITICAL
**Ventricular tachycardia**												
16	randomised trials	no serious risk of bias	no serious inconsistency	no serious indirectness	no serious imprecision	none	179/43415 (0.41%)	166/36628 (0.45%)	RR 0.99 (0.80 to 1.22)	0 fewer per 1000 (from 1 more to 1 more)	⊕⊕⊕⊕ HIGH	CRITICAL
**Ventricular extrasystoles**												
10	randomised trials	no serious risk of bias	no serious inconsistency	no serious indirectness	serious[1]	none	24/39867 (0.06%)	14/33305 (0.04%)	RR 1.36 (0.67 to 2.76)	0 more per 1000 (from 0 fewer to 1 more)	⊕⊕⊕O MODERATE	IMPORTANT
**Sudden cardiac death**												
13	randomised trials	no serious risk of bias	no serious inconsistency	no serious indirectness	serious[1]	none	88/42695 (0.21%)	96/36037 (0.27%)	RR 0.85 (0.63 to 1.14)	0 fewer per 1000 (from 1 fewer to 0 more)	⊕⊕⊕O MODERATE	CRITICAL
**VF/VT/SCD**												
19	randomised trials	no serious risk of bias	no serious inconsistency	no serious indirectness	no serious imprecision	none	320/44053 (0.73%)	307/37077 (0.83%)	RR 0.95 (0.81 to 1.11)	0 fewer per 1000 (from 2 fewer to 1 more)	⊕⊕⊕⊕ HIGH	CRITICAL

[1] Imprecision was downgraded because the 95% of the relative risk was sufficiently wide that the estimate could include appreciable harm or benefit of the intervention (thresholds: 0.75 and 1.25).
