# Supplementary material for: Effect of SGLT-2 inhibitors on arrhythmia events: insight from an updated secondary analysis of > 80,000 patients (the SGLT2i—Arrhythmias and Sudden Cardiac Death)
Source: Cardiovasc Diabetol. 2024 Feb 24;23:78. doi: 10.1186/s12933-024-02137-x (PMC10893620; doi:10.1186/s12933-024-02137-x)
Supplement: Supplementary file 2 — Additional file 2. Additional references. [file 12933_2024_2137_MOESM2_ESM.pdf]

Because of word count and reference number limitation we list the Supplementary References (included RCTs) here:

1. Inagaki N, Kondo K, Yoshinari T, Maruyama N, Susuta Y, Kuki H. Efficacy and safety of canagliflozin in Japanese patients with type 2 diabetes: a randomized, double-blind, placebo-controlled, 12-week study. *DIABETES OBES METAB* 2013; 15(12):1136-1145.
2. Wilding JP, Charpentier G, Hollander P, González-Gálvez G, Mathieu C, Vercruysse F, Usiskin K, Law G, Black S, Canovatchel W, Meininger G. Efficacy and safety of canagliflozin in patients with type 2 diabetes mellitus inadequately controlled with metformin and sulphonylurea: a randomised trial. *INT J CLIN PRACT* 2013; 67(12):1267-1282.
3. Yale JF, Bakris G, Cariou B, Nieto J, David-Neto E, Yue D, Wajs E, Figueroa K, Jiang J, Law G, Usiskin K, Meininger G. Efficacy and safety of canagliflozin over 52 weeks in patients with type 2 diabetes mellitus and chronic kidney disease. *DIABETES OBES METAB* 2014; 16(10):1016-1027.
4. Bode B, Stenlöf K, Harris S, Sullivan D, Fung A, Usiskin K, Meininger G. Long-term efficacy and safety of canagliflozin over 104 weeks in patients aged 55-80 years with type 2 diabetes. *DIABETES OBES METAB* 2015; 17(3):294-303.
5. Neal B, Perkovic V, Mahaffey KW, de Zeeuw D, Fulcher G, Erondu N, Shaw W, Law G, Desai M, Matthews DR. Canagliflozin and Cardiovascular and Renal Events in Type 2 Diabetes. *NEW ENGL J MED* 2017; 377(7):644-657.
6. Perkovic V, Jardine MJ, Neal B, Bompoint S, Heerspink H, Charytan DM, Edwards R, Agarwal R, Bakris G, Bull S, Cannon CP, Capuano G, Chu PL, de Zeeuw D, Greene T, Levin A, Pollock C, Wheeler DC, Yavin Y, Zhang H, Zinman B, Meininger G, Brenner BM, Mahaffey KW. Canagliflozin and Renal Outcomes in Type 2 Diabetes and Nephropathy. *The New England journal of medicine* 2019; 380(24):2295-2306.
7. Wilding JP, Woo V, Soler NG, Pahor A, Sugg J, Rohwedder K, Parikh S. Long-term efficacy of dapagliflozin in patients with type 2 diabetes mellitus receiving high doses of insulin: a randomized trial. *ANN INTERN MED* 2012; 156(6):405-415.
8. Bailey CJ, Gross JL, Hennicken D, Iqbal N, Mansfield TA, List JF. Dapagliflozin add-on to metformin in type 2 diabetes inadequately controlled with metformin: a randomized, double-blind, placebo-controlled 102-week trial. *BMC MED* 2013; 11:43.
9. Leiter LA, Cefalu WT, de Bruin TW, Gause-Nilsson I, Sugg J, Parikh SJ. Dapagliflozin added to usual care in individuals with type 2 diabetes mellitus with preexisting cardiovascular disease: a 24-week, multicenter, randomized, double-blind, placebo-controlled study with a 28-week extension. *J AM GERIATR SOC* 2014; 62(7):1252-1262.
10. Cefalu WT, Leiter LA, de Bruin TW, Gause-Nilsson I, Sugg J, Parikh SJ. Dapagliflozin's Effects on Glycemia and Cardiovascular Risk Factors in High-Risk Patients With Type 2 Diabetes: A 24-Week, Multicenter, Randomized, Double-Blind, Placebo-Controlled Study With a 28-Week Extension. *DIABETES CARE* 2015; 38(7):1218-1227.
11. Nassif ME, Windsor SL, Tang F, Khariton Y, Husain M, Inzucchi SE, McGuire DK, Pitt B, Scirica BM, Austin B, Drazner MH, Fong MW, Givertz MM, Gordon RA, Jermyn R, Katz SD, Lamba S, Lanfear DE, LaRue SJ, Lindenfeld J, Malone M, Margulies K, Mentz RJ, Mutharasan RK, Pursley M, Umpierrez G, Kosiborod M. Dapagliflozin Effects on Biomarkers, Symptoms, and Functional Status in Patients With Heart Failure With Reduced Ejection Fraction: The DEFINE-HF Trial. *CIRCULATION* 2019; 140(18):1463-1476.
12. McMurray J, Solomon SD, Inzucchi SE, Køber L, Kosiborod MN, Martinez FA, Ponikowski P,

Sabatine MS, Anand IS, Bělohlávek J, Böhm M, Chiang CE, Chopra VK, de Boer RA, Desai AS, Diez M, Drozdz J, Dukát A, Ge J, Howlett JG, Katova T, Kitakaze M, Ljungman C, Merkely B, Nicolau JC, O'Meara E, Petrie MC, Vinh PN, Schou M, Tereshchenko S, Verma S, Held C, DeMets DL, Docherty KF, Jhund PS, Bengtsson O, Sjöstrand M, Langkilde AM. Dapagliflozin in Patients with Heart Failure and Reduced Ejection Fraction. *The New England journal of medicine* 2019; 381(21):1995-2008.

13. Wiviott SD, Raz I, Bonaca MP, Mosenzon O, Kato ET, Cahn A, Silverman MG, Zelniker TA, Kuder JF, Murphy SA, Bhatt DL, Leiter LA, McGuire DK, Wilding J, Ruff CT, Gause-Nilsson I, Fredriksson M, Johansson PA, Langkilde AM, Sabatine MS. Dapagliflozin and Cardiovascular Outcomes in Type 2 Diabetes. *The New England journal of medicine* 2019; 380(4):347-357.

14. Heerspink H, Stefánsson BV, Correa-Rotter R, Chertow GM, Greene T, Hou FF, Mann J, McMurray J, Lindberg M, Rossing P, Sjöström CD, Toto RD, Langkilde AM, Wheeler DC. Dapagliflozin in Patients with Chronic Kidney Disease. *The New England journal of medicine* 2020; 383(15):1436-1446.

15. Barnett AH, Mithal A, Manassie J, Jones R, Rattunde H, Woerle HJ, Broedl UC. Efficacy and safety of empagliflozin added to existing antidiabetes treatment in patients with type 2 diabetes and chronic kidney disease: a randomised, double-blind, placebo-controlled trial. *LANCET DIABETES ENDO* 2014; 2(5):369-384.

16. Rosenstock J, Jelaska A, Zeller C, Kim G, Broedl UC, Woerle HJ. Impact of empagliflozin added on to basal insulin in type 2 diabetes inadequately controlled on basal insulin: a 78-week randomized, double-blind, placebo-controlled trial. *DIABETES OBES METAB* 2015; 17(10):936-948.

17. Kovacs CS, Seshiah V, Merker L, Christiansen AV, Roux F, Salsali A, Kim G, Stella P, Woerle HJ, Broedl UC. Empagliflozin as Add-on Therapy to Pioglitazone With or Without Metformin in Patients With Type 2 Diabetes Mellitus. *CLIN THER* 2015; 37(8):1773-1788.

18. Roden M, Merker L, Christiansen AV, Roux F, Salsali A, Kim G, Stella P, Woerle HJ, Broedl UC. Safety, tolerability and effects on cardiometabolic risk factors of empagliflozin monotherapy in drug-naïve patients with type 2 diabetes: a double-blind extension of a Phase III randomized controlled trial. *CARDIOVASC DIABETOL* 2015; 14:154.

19. Zinman B, Wanner C, Lachin JM, Fitchett D, Bluhmki E, Hantel S, Mattheus M, Devins T, Johansen OE, Woerle HJ, Broedl UC, Inzucchi SE. Empagliflozin, Cardiovascular Outcomes, and Mortality in Type 2 Diabetes. *NEW ENGL J MED* 2015; 373(22):2117-2128.

20. Søfteland E, Meier JJ, Vangen B, Toorawa R, Maldonado-Lutomirsky M, Broedl UC. Empagliflozin as Add-on Therapy in Patients With Type 2 Diabetes Inadequately Controlled With Linagliptin and Metformin: A 24-Week Randomized, Double-Blind, Parallel-Group Trial. *DIABETES CARE* 2017; 40(2):201-209.

21. Verma S, Mazer CD, Yan AT, Mason T, Garg V, Teoh H, Zuo F, Quan A, Farkouh ME, Fitchett DH, Goodman SG, Goldenberg RM, Al-Omran M, Gilbert RE, Bhatt DL, Leiter LA, Jüni P, Zinman B, Connelly KA. Effect of Empagliflozin on Left Ventricular Mass in Patients With Type 2 Diabetes Mellitus and Coronary Artery Disease: The EMPA-HEART CardioLink-6 Randomized Clinical Trial. *CIRCULATION* 2019; 140(21):1693-1702.

22. Damman K, Beusekamp JC, Boorsma EM, Swart HP, Smilde T, Elvan A, van Eck J, Heerspink H, Voors AA. Randomized, double-blind, placebo-controlled, multicentre pilot study on the effects of empagliflozin on clinical outcomes in patients with acute decompensated heart failure (EMPA-RESPONSE-AHF). *EUR J HEART FAIL* 2020; 22(4):713-722.

23. Santos-Gallego CG, Vargas-Delgado AP, Requena-Ibanez JA, Garcia-Ropero A, Mancini D,

Pinney S, Macaluso F, Sartori S, Roque M, Sabatel-Perez F, Rodriguez-Cordero A, Zafar MU, Fergus I, Atallah-Lajam F, Contreras JP, Varley C, Moreno PR, Abascal VM, Lala A, Tamler R, Sanz J, Fuster V, Badimon JJ. Randomized Trial of Empagliflozin in Nondiabetic Patients With Heart Failure and Reduced Ejection Fraction. *J AM COLL CARDIOL* 2021; 77(3):243-255.
24. Abraham WT, Lindenfeld J, Ponikowski P, Agostoni P, Butler J, Desai AS, Filippatos G, Gniot J, Fu M, Gullestad L, Howlett JG, Nicholls SJ, Redon J, Schenkenberger I, Silva-Cardoso J, Störk S, Krzysztof WJ, Savarese G, Brueckmann M, Jamal W, Nordaby M, Peil B, Ritter I, Ustyugova A, Zeller C, Salsali A, Anker SD. Effect of empagliflozin on exercise ability and symptoms in heart failure patients with reduced and preserved ejection fraction, with and without type 2 diabetes. *EUR HEART J* 2021; 42(6):700-710.
25. Packer M, Anker SD, Butler J, Filippatos G, Pocock SJ, Carson P, Januzzi J, Verma S, Tsutsui H, Brueckmann M, Jamal W, Kimura K, Schnee J, Zeller C, Cotton D, Bocchi E, Böhm M, Choi DJ, Chopra V, Chuquiure E, Giannetti N, Janssens S, Zhang J, Gonzalez Juanatey JR, Kaul S, Brunner-La Rocca HP, Merkely B, Nicholls SJ, Perrone S, Pina I, Ponikowski P, Sattar N, Senni M, Seronde MF, Spinar J, Squire I, Taddei S, Wanner C, Zannad F. Cardiovascular and Renal Outcomes with Empagliflozin in Heart Failure. *The New England journal of medicine* 2020; 383(15):1413-1424.
26. Anker SD, Butler J, Filippatos G, Ferreira JP, Bocchi E, Böhm M, Brunner-La Rocca HP, Choi DJ, Chopra V, Chuquiure-Valenzuela E, Giannetti N, Gomez-Mesa JE, Janssens S, Januzzi JL, Gonzalez-Juanatey JR, Merkely B, Nicholls SJ, Perrone SV, Piña IL, Ponikowski P, Senni M, Sim D, Spinar J, Squire I, Taddei S, Tsutsui H, Verma S, Vinereanu D, Zhang J, Carson P, Lam C, Marx N, Zeller C, Sattar N, Jamal W, Schnaidt S, Schnee JM, Brueckmann M, Pocock SJ, Zannad F, Packer M. Empagliflozin in Heart Failure with a Preserved Ejection Fraction. *The New England journal of medicine* 2021; 385(16):1451-1461.
27. Voors AA, Angermann CE, Teerlink JR, Collins SP, Kosiborod M, Biegus J, Ferreira JP, Nassif ME, Psotka MA, Tromp J, Borleffs C, Ma C, Comin-Colet J, Fu M, Janssens SP, Kiss RG, Mentz RJ, Sakata Y, Schirmer H, Schou M, Schulze PC, Spinarova L, Volterrani M, Wranicz JK, Zeymer U, Zieroth S, Brueckmann M, Blatchford JP, Salsali A, Ponikowski P. The SGLT2 inhibitor empagliflozin in patients hospitalized for acute heart failure: a multinational randomized trial. *NAT MED* 2022; 28(3):568-574.
28. Rosenstock J, Frias J, Páll D, Charbonnel B, Pascu R, Saur D, Darekar A, Huyck S, Shi H, Lauring B, Terra SG. Effect of ertugliflozin on glucose control, body weight, blood pressure and bone density in type 2 diabetes mellitus inadequately controlled on metformin monotherapy (VERTIS MET). *DIABETES OBES METAB* 2018; 20(3):520-529.
29. Grunberger G, Camp S, Johnson J, Huyck S, Terra SG, Mancuso JP, Jiang ZW, Golm G, Engel SS, Lauring B. Ertugliflozin in Patients with Stage 3 Chronic Kidney Disease and Type 2 Diabetes Mellitus: The VERTIS RENAL Randomized Study. *DIABETES THER* 2018; 9(1):49-66.
30. Cannon CP, Pratley R, Dagogo-Jack S, Mancuso J, Huyck S, Masiukiewicz U, Charbonnel B, Frederich R, Gallo S, Cosentino F, Shih WJ, Gantz I, Terra SG, Cherney D, McGuire DK. Cardiovascular Outcomes with Ertugliflozin in Type 2 Diabetes. *The New England journal of medicine* 2020; 383(15):1425-1435.
31. Bhatt DL, Szarek M, Pitt B, Cannon CP, Leiter LA, McGuire DK, Lewis JB, Riddle MC, Inzucchi SE, Kosiborod MN, Cherney D, Dwyer JP, Scirica BM, Bailey CJ, Díaz R, Ray KK, Udell JA, Lopes RD, Lapuerta P, Steg PG. Sotagliflozin in Patients with Diabetes and Chronic Kidney Disease. *The New England journal of medicine* 2021; 384(2):129-139.
32. Bhatt DL, Szarek M, Steg PG, Cannon CP, Leiter LA, McGuire DK, Lewis JB, Riddle MC, Voors

AA, Metra M, Lund LH, Komajda M, Testani JM, Wilcox CS, Ponikowski P, Lopes RD, Verma S, Lapuerta P, Pitt B. Sotagliflozin in Patients with Diabetes and Recent Worsening Heart Failure. *The New England journal of medicine* 2021; 384(2):117-128.
